# ﻿Next step in *Monachacantiana* (Montagu, 1803) phylogeography: northern French and Dutch populations (Eupulmonata, Stylommatophora, Hygromiidae)

**DOI:** 10.3897/zookeys.1198.119738

**Published:** 2024-04-23

**Authors:** Joanna R. Pieńkowska, Giuseppe Manganelli, Małgorzata Proćków, Debora Barbato, Katarzyna Sosnowska, Folco Giusti, Andrzej Lesicki

**Affiliations:** 1 Department of Cell Biology, Institute of Experimental Biology, Faculty of Biology, Adam Mickiewicz University in Poznań, Uniwersytetu Poznańskiego 6, 61-614 Poznań, Poland Adam Mickiewicz University in Poznań Poznań Poland; 2 Dipartimento di Scienze Fisiche, della Terra e dell’Ambiente, Università di Siena, Via Mattioli 4, 53100 Siena, Italy Università di Siena Siena Italy; 3 NBFC (National Biodiversity Future Center), Palermo, Italy NBFC (National Biodiversity Future Center) Palermo Italy; 4 Museum of Natural History, University of Wrocław, Sienkiewicza 21, 50-335 Wrocław, Poland University of Wrocław Wrocław Poland

**Keywords:** 16SrDNA, COI, genitalia, H3, ITS2, mitochondrial and nuclear genes, nucleotide sequences, population distribution, shell

## Abstract

Features of shell and genitalia as well as nucleotide sequences of selected mitochondrial and nuclear genes of specimens of *Monachacantiana* from ten northern French and two Dutch populations were compared with the same features of British and Italian populations. They were found to be very similar to populations previously identified as belonging to the CAN-1 lineage of *M.cantiana*. This confirms previous suggestions that *M.cantiana* was introduced to western Europe (England, France and the Netherlands) in historical times.

## ﻿Introduction

*Monacha* Fitzinger, 1833 is a species-rich genus including numerous nominal species diversified mainly in the Anatolian and European parts of Turkey, in the southern parts of the Balkans and in Italy (Hausdorf 2000a, 2000b; Welter-Schultes 2012; Neiber and Hausdorf 2017). Only two species, *Monachacantiana* (Montagu, 1803) and *M.cartusiana* (Müller, 1774), used to be reported from Western Europe. Two more were introduced not long ago, namely *M.ocellata* (Roth, 1839) and *M.samsunensis* (Pfeiffer, 1868), the latter until recently reported as *M.atacis* Gittenberger & de Winter, 1985 (Welter-Schultes 2012; Anderson et al. 2018; Pieńkowska et al. 2018a, 2022).

*Monachacantiana*, commonly known as the Kentish snail, was described by Montagu (1803: 422) from Kent in Britain “where it is found chiefly upon the chalky soil”. Type material consists of three syntypes, which were probably collected around Sandwich in Kent (51°16'26.46"N, 1°20'14.74"E) by William Boys, and are kept with the Montagu Collection in the Royal Albert Memorial Museum & Art Gallery, Exeter (Oliver et al. 2017). Montagu later added several localities in other counties of southern Britain to the original description (Montagu 1808: 145, pl. 23, fig. 1).

**Figure 1.**
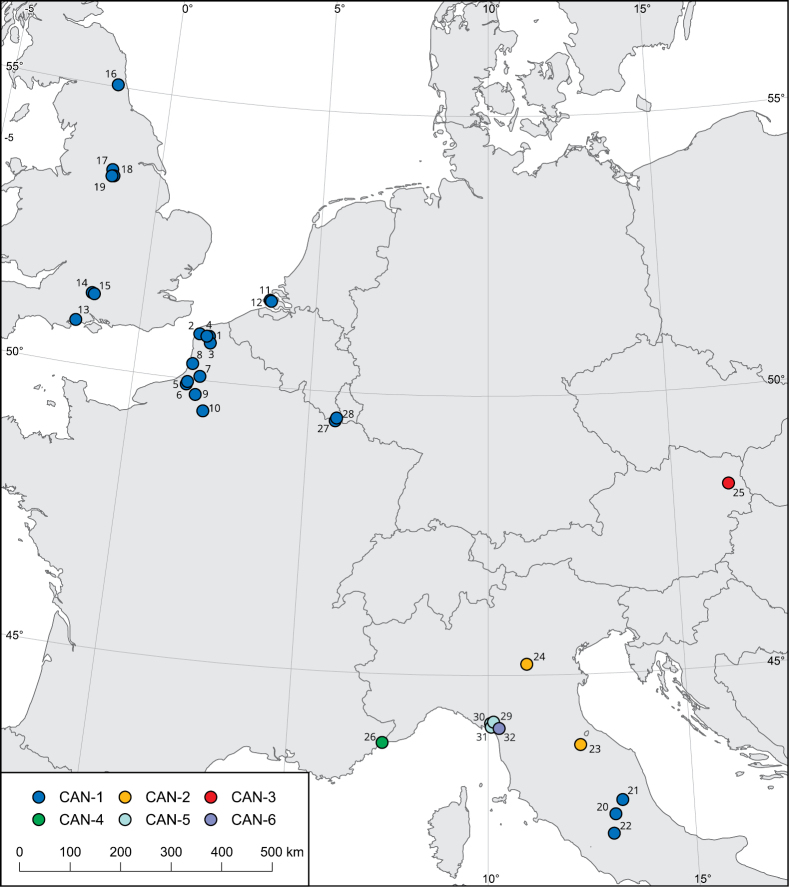
Map of localities of the populations of *Monachacantiana* analysed. See Table 1 for details of populations 1–26, Brulé and Bichain (2019) for populations 27 and 28, and Pieńkowska et al. (2019a) for populations 29–32.

It has been suggested that this species was introduced to the British Isles in historical times (Kerney 1970, 1999; Evans 1972). Our previous research on several *M.cantiana* populations, using an integrative approach combining analysis of the shell structure and genital anatomy with that of nucleotide sequences of mitochondrial and nuclear gene fragments, revealed six lineages, namely CAN-1, CAN-2, CAN-3, CAN-4, CAN-5 and CAN-6 (Pieńkowska et al. 2018b, 2019a). CAN-1 (representing true *M.cantiana*) was found to occur in the Latium region of Italy and in Spain and Britain (Pieńkowska et al. 2018b; Čejka et al. 2020), in line with the suggestion that this lineage probably spread with the Roman conquests (Pieńkowska et al. 2018b). Populations of CAN-2 were found in regions of Italy (Emilia Romagna) north of Latium (Pieńkowska et al. 2018b) and somewhat surprisingly in Slovakia (Bratislava) (Čejka et al. 2022), while those of CAN-3 were reportedly widespread even further north in Italy (Friuli-Venezia Giulia) as far as Vienna in Austria (Pieńkowska et al. 2018b, 2019b) and Bratislava in Slovakia (Čejka et al. 2022). The lineage CAN-4, corresponding to *Monachacemenelea* (Risso, 1826), was found in south-eastern France (Pieńkowska et al. 2018b; Čejka et al. 2020). CAN-5 and CAN-6 are reported from the Apuan Alps and represent one or two different species, the naming of which requires further studies on topotypical material (Pieńkowska et al. 2019a).

*Monachacantiana* has been reported from France (Kerney et al. 1983; Falkner et al. 2002; Cucherat 2005; Lecaplain 2007; Gargominy et al. 2011; Welter-Schultes 2012; Bichain et al. 2019; Brulé and Bichain 2019; INPN 2019). Brulé and Bichain (2019) carefully analysed shell and genitalia features of *M.cantiana* specimens collected at two sites in north-eastern France near the towns of Cutry and Longwy. However since the CAN-1, CAN-2, CAN-3, and CAN-4 lineages of *M.cantiana* do not differ in shell or genital features, the phylogenetic relationships of populations from north-eastern France had to be clarified by genetic analysis. Although *M.cantiana* is known to occur in the Netherlands (Kerney et al. 1983; Gittenberger et al. 1984; Welter-Schultes 2012), it has never been confirmed genetically.

The aim of the present research was: 1) to study morphological (shell and genitalia) and molecular variation in specimens of *M.cantiana* collected in northern France and the Netherlands in order to clarify their relations to the British and Italian populations; 2) to test the hypothesis that the English, French and Dutch populations originated from the same introduced propagules.

## ﻿Materials and methods

### ﻿Taxonomic samples

Specimens from ten French and two Dutch populations of *Monachacantiana* were considered for analysis of the variability of their molecular and morphological (shell and genitalia) features (Table 1, Fig. 1). Specimens from four new British and one new Italian population were used for comparative molecular analysis with other populations of *M.cantiana* s.l. (Table 1, Fig. 1). Sequences deposited in GenBank for *M.cantiana* s.l. from other populations (Manganelli et al. 2005; Duda et al. 2011; Kruckenhauser et al. 2014; Cadahia et al. 2014; Pieńkowska et al. 2015, 2018b, 2019a, 2019b; Razkin et al. 2015; Neiber and Hausdorf 2017; Čejka et al. 2020, 2022) and three other *Monacha* species (*M.cartusiana*: Pieńkowska et al. 2015, 2022; Neiber and Hausdorf 2017; Caro et al. 2019; Čejka et al. 2020; *M.pantanellii* (De Stefani, 1879): Pieńkowska et al. 2020; *M.parumcincta* (Rossmässler, 1834): Pieńkowska et al. 2018b) were also selected for molecular analysis (Suppl. materials 1–4) and supplemented with several new sequences of mitochondrial (16SrDNA) and nuclear (ITS2 flanked with 5.8SrDNA and 28SrDNA) genes (Table 1). Sequences of *Trochulushispidus* (Linnaeus, 1758) deposited in GenBank by Neiber et al. (2017), Neiber and Hausdorf (2017), Caro et al. (2019) and Proćków et al. (2021) were used as an outgroup to construct phylogenetic trees (Suppl. materials 1–4). The localities for reference populations of *M.cantiana* s.l. CAN-1 – CAN-6, *M.pantanellii*, *M.cartusiana*, and *M.parumcincta* were shown on maps published in our previous papers (Pieńkowska et al. 2018b: fig. 63, 2020: fig. 1).

**Table 1.** List of localities of *Monachacantiana* s.l. populations used for molecular and morphological (SH shell, AN genitalia) research.

Localities				Current taxonomy	Clade	Designation of DNA voucher sps	COI		Long 16SrDNA		H3		5.8SrDNA + ITS2 + 28SrDNA		PCA and RDA	Figs
No.	coordinates	country and site	collector / date / no. of specimens (collection)				new haplotype	GenBank ##	new haplotype	GenBank ##	new haplotype	GenBank ##	new haplotype	GenBank ##		
1	50°47'56.7"N, 02°00'57.5"E	France, Pas-de-Calais, Bonningues-lès-Ardres, vegetation under shrubs, 42 m a.s.l.	M. Proćków / 20.06.2018 / 5 (MNHW* F.18.13)	*M.cantiana*	CAN-1	Ard1			16S 1	OR918363	H3 1	OR939858	ITS2 1	OR917347	AN	
						Ard2	COI 1	OR918493	16S 1	OR918364	H3 2	OR939859	ITS2 2	OR917348	AN	
						Ard3			16S 1	OR918365	H3 1	OR939860	ITS2 1	OR917349		
						Ard4	COI 1	OR918494	16S 2	OR918366	H3 1	OR939861	ITS2 1	OR917350		
						Ard5					H3 3	OR939862				
2	50°49'28.1"N, 01°44'01.9"E	France, Pas-de-Calais, Blecquenecques n. Marquise, roadside, 26 m a.s.l.	M. Proćków / 20.06.2018 / 5 (MNHW F.18.10)	*M.cantiana*	CAN-1	Ble1	COI 1	OR918495	16S 3	OR918367	H3 1	OR939863			SH/AN	SH/AN
						Ble2	COI 1	OR918496	16S 1	OR918368	H3 1	OR939864	ITS2 3	OR917351		
						Ble4	COI 1	OR918497	16S 3	OR918369	H3 1	OR939865				
						Ble5	COI 1	OR918498			H3 1	OR939866	ITS2 4	OR917352		
3	50°40'56.7"N, 02°03'39.1"E	France, Pas-de-Calais, Larré, vegetation along stream, 65 m a.s.l.	M. Proćków / 20.06.2018 / 5 (MNHW F.18.14)	*M.cantiana*	CAN-1	Lar1	COI 1	OR918499	16S 3	OR918370	H3 3	OR939867	ITS2 5	OR917353		
						Lar2	COI 2	OR918500	16S 4	OR918371	H3 1	OR939868	ITS2 6	OR917354		
						Lar3	COI 1	OR918501	16S 4	OR918372	H3 1	OR939869	ITS2 1	OR917355		
						Lar4	COI 3	OR918502			H3 4	OR939870	ITS2 7	OR917356		
						Lar5	COI 1	OR918503	16S 4	OR918373	H3 1	OR939871	ITS2 7	OR917357		
4	50°47'48.2"N, 01°56'34.4"E	France, Pas-de-Calais, Licques, vegetation along road, 81 m a.s.l.	M. Proćków / 20.06.2018 / 5 (MNHW F.18.12)	*M.cantiana*	CAN-1	Lic1	COI 1	OR918504	16S 1	OR918374	H3 1	OR939872	ITS2 1	OR917358		
						Lic2	COI 1	OR918505	16S 3	OR918375	H3 1	OR939873	ITS2 8	OR917359		
						Lic3			16S 5	OR918376	H3 3	OR939874	ITS2 1	OR917360		
						Lic4	COI 1	OR918506	16S 5	OR918377	H3 1	OR939875	ITS2 9	OR917361		
						Lic5	COI 1	OR918507	16S 1	OR918378	H3 1	OR939876	ITS2 1	OR917362		
5	49°54'23.6"N, 01°30'58.9"E	France, Seine-Maritime, Béthencourt n. Grandcourt, vegetation under trees, 97 m a.s.l.	M. Proćków / 23.06.2018 / 5 (MNHW F.18.22)	*M.cantiana*	CAN-1	Bet1	COI 1	OR918508	16S 3	OR918379	H3 1	OR939877	ITS2 10	OR917363	SH/AN	SH/AN
						Bet2	COI 1	OR918509			H3 1	OR939878	ITS2 11	OR917364		
						Bet3			16S 6	OR918380	H3 1	OR939879				
						Bet4	COI 1	OR918510	16S 6	OR918381	H3 1	OR939880	ITS2 12	OR917365		
						Bet5	COI 1	OR918511	16S 3	OR918382	H3 1	OR939881	ITS2 13	OR917366		
6	49°55'05.6"N, 01°31'38.1"E	France, Seine-Maritime, Pierrepont, forest edge, 146 m a.s.l.	M. Proćków / 23.06.2018 / 5 (MNHW F.18.21)	*M.cantiana*	CAN-1	Pie1	COI 1	OR918512	16S 3	OR918383	H3 1	OR939882	ITS2 14	OR917367	SH/AN	SH/AN
						Pie2	COI 1	OR918513			H3 1	OR939883	ITS2 1	OR917368		
						Pie3	COI 1	OR918514			H3 1	OR939884				
						Pie4	COI 1	OR918515	16S 3	OR918384	H3 1	OR939885	ITS2 15	OR917369		
7	50°04'05.1"N, 01°52'20.9"E	France, Somme, Épagne-Épagnette, roadside, 13 m a.s.l.	M. Proćków / 19.06.2018 / 5 (MNHW F.18.08)	*M.cantiana*	CAN-1	Epa1	COI 1	OR918516	16S 7	OR918385	H3 3	OR939886	ITS2 16	OR917370	SH/AN	AN
						Epa2	COI 1	OR918517	16S 3	OR918386	H3 1	OR939887	ITS2 1	OR917371		
						Epa3	COI 4	OR918518			H3 1	OR939888	ITS2 17	OR917372		
						Epa4			16S 8	OR918387	H3 5	OR939889	ITS2 18	OR917373		
						Epa5	COI 1	OR918519	16S 9	OR918388	H3 1	OR939890				
8	50°16'54.7"N, 01°37'41.9"E	France, Somme, Froise, forest edge, 86 m a.s.l.	M. Proćków / 19.06.2018 / 5 (MNHW F.18.20)	*M.cantiana*	CAN-1	Fro1			16S 4	OR918389	H3 2	OR939891				
						Fro2	COI 1	OR918520	16S 10	OR918390	H3 1	OR939892	ITS2 19	OR917374		
						Fro3	COI 1	OR918521	16S 11	OR918391	H3 1	OR939893	ITS2 1	OR917375		
						Fro4	COI 1	OR918522	16S 12	OR918392	H3 1	OR939894				
						Fro5	COI 1	OR918523	16S 13	OR918393	H3 2	OR939895				
9	49°44'14.7"N, 01°47'53.9"E	France, Oise, Escales-Saint-Pierre, roadside, 164 m a.s.l.	M. Proćków / 19.06.2018 / 5 (MNHW F.18.06)	*M.cantiana*	CAN-1	Esc1	COI 1	OR918524	16S 14	OR918394	H3 1	OR939896	ITS2 20	OR917376	SH/AN	SH/AN
						Esc2	COI 1	OR918525			H3 2	OR939897	ITS2 21	OR917377		
						Esc3	COI 1	OR918526	16S 14	OR918395	H3 6	OR939898	ITS2 22	OR917378		
						Esc4	COI 1	OR918527			H3 1	OR939899	ITS2 23	OR917379		
						Esc5	COI 1	OR918528	16S 15	OR918396	H3 6	OR939900	ITS2 17	OR917380		
10	49°27'38.2"N, 02°03'35.0"E	France, Oise, Fouquenies, vegetation along forest road, 29 m a.s.l.	M. Proćków / 19.06.2018 / 5 (MNHW F.18.05)	*M.cantiana*	CAN-1	Fou1	COI 1	OR918529	16S 3	OR918397	H3 3	OR939901	ITS2 24	OR917381		
						Fou2	COI 5	OR918530	16S 16	OR918398	H3 1	OR939902	ITS2 1	OR917382		
						Fou3			16S 3	OR918399	H3 7	OR939903				
						Fou4			16S 17	OR918400	H3 7	OR939904	ITS2 25	OR917383		
						Fou5	COI 1	OR918531	16S 18	OR918401	H3 1	OR939905	ITS2 26	OR917384		
11	51°32'57.0"N,, 03°39'27.9"E	The Netherlands, Veere, edge of forest, 15 m a.s.l.	M. Proćków / 6.06.2019/ 5 (MNHW NL.19.02)	*M.cantiana*	CAN-1	Vee1-1	COI 1	OR918532	16S 19	OR918402	H3 1	OR939906				
						Vee1-2	COI 1	OR918533	16S 1	OR918403	H3 8	OR939907				
						Vee1-3	COI 1	OR918534	16S 3	OR918404	H3 1	OR939908				
						Vee1-4	COI 1	OR918535	16S 19	OR918405	H3 1	OR939909				
						Vee1-5	COI 6	OR918536	16S 3	OR918406	H3 1	OR939910				
12	51°32'57.1"N,, 03°39'40.1"E	The Netherlands, Veere 6, vegetation near windmill, 81 m a.s.l.	M. Proćków / 7.06.2019/ 5 (MNHW NL.19.07)	*M.cantiana*	CAN-1	Vee2-1			16S 3	OR918407	H3 1	OR939911				
						Vee2-2	COI 7	OR918537	16S 3	OR918408	H3 5	OR939912				
						Vee2-3	COI 1	OR918538	16S 19	OR918409	H3 1	OR939913				
						Vee2-4	COI 1	OR918539	16S 3	OR918410	H3 1	OR939914				
						Vee2-5		OR918540	16S 3	OR918411	H3 1	OR939915				
13.	50°46'23.5"N, 01°50'06.3"W	United Kingdom, Hurn, vegetation along road, 7 m a.s.l.	M. Proćków / 15.06.2022/ 2 (MNHW GB.22.04)	*M.cantiana*	CAN-1	Hum1			16S 1	OR918412	H3 1	OR939916	ITS2 1	OR917385		
						Hum2	COI 8	OR918541			H3 9	OR939917				
14.	51°17'43.7"N, 01°29'34.9"W	United Kingdom, Vernhams Dean, vegetation along shaded path, 136 m a.s.l.	M. Proćków / 15.06.2022/ 4 (MNHW GB.22.05)	*M.cantiana*	CAN-1	Ver1			16S 3	OR918413	H3 9	OR939918	ITS2 1	OR917386		
						Ver2			16S 3	OR918414	H3 9	OR939919	ITS2 1	OR917387		
						Ver3					H3 10	OR939920	ITS2 27	OR917388		
						Ver4			16S 3	OR918415	H3 1	OR939921	ITS2 1	OR917389		
15.	51°17'32.3"N, 01°29'10.9"W	United Kingdom, Upton, vegetation along road, 120 m a.s.l.	M. Proćków / 15.06.2022/ 2 (MNHW GB.22.06)	*M.cantiana*	CAN-1	Upt1			16S 3	OR918416	H3 1	OR939922	ITS2 1	OR917390		
						Upt2			16S 3	OR918417	H3 1	OR939923	ITS2 1	OR917391		
16.	55°02'13.6"N, 01°42'51.0"W	United Kingdom, Newcastle upon Tyne, vegetation near airport, 80 m a.s.l.	M. Proćków / 15.06.2022/ 6 (MNHW GB.22.07)	*M.cantiana*	CAN-1	New1	COI 9	OR918542	16S 20	OR918418	H3 9	OR939924	ITS2 1	OR917392		
						New2			16S 20	OR918419	H3 9	OR939925	ITS2 1	OR917393		
						New3	COI 10	OR918543	16S 20	OR918420	H3 9	OR939926	ITS2 1	OR917394		
						New4	COI 9	OR918544	16S 20	OR918421	H3 1	OR939927	ITS2 1	OR917395		
						New5	COI 1	OR918545	16S 3	OR918422	H3 9	OR939928	ITS2 1	OR917396		
						New6	COI 9	OR918546	16S 20	OR918423	H3 1	OR939929	ITS2 1	OR917397		
17.	53°31'29"N, 01°27'54"W	United Kingdom, Barrow near Barnsley	R.A.D. Cameron / 10.2011 / 5 (FGC* 40329)	*M.cantiana*	CAN-1	8FG-1		MG208884	16S 1	OR918424		MG209031	ITS2 1	OR917398		
						8FG-2		MG208885	16S 1	OR918425		MG209032	ITS2 1	OR917399		
18.	53°25'04.2"N, 01°24'00.5"W	United Kingdom, Rotherham	R.A.D. Cameron / 07.2015 / 7 (DCBC*)	*M.cantiana*	CAN-1	Sit1-1		MG208893	16S 1	OR918426		MG209035	ITS2 28	OR917400		
19.	53°24'49.1"N, 01°24'36.6"W	United Kingdom, Sheffield	R.A.D. Cameron / 07.2015 / 6 (DCBC)	*M.cantiana*	CAN-1	Sit2-1		MG208899	16S 21	OR918427		MG209038	ITS2 1	OR917401		
20	42°28'41.05"N, 13°05'09.46"E	Italy, Latium, Gole del Velino, near Sigillo (Posta, Rieti)	A. Hallgass / 30.09.2012 / 8 (FGC 42960)	*M.cantiana*	CAN-1	4FG-1		MG208905	16S 24	OR918428		MG209039	ITS2 29	OR917402		
						4FG-2		MG208910	16S 25	OR918429		MG209042	ITS2 29	OR917403		
21.	42°43'39.87"N, 13°16'01.44"E	Italy, Latium, Valle del Tronto (Accumoli, Rieti)	A. Hallgass / 30.09.2012 / 4 (FGC 42963)	*M.cantiana*	CAN-1	Tro1		MG208921	16S 26	OR918430		MG209043	ITS2 1	OR917404		
22.	42°07'53.39"N, 13°01'39.81"E	Italy, Latium, Valle del Turano, near Turania (Rieti)	A. Hallgass / 04.11.2013 / 2 (FGC 42969)	*M.cantiana*	CAN-1	Tur5-1		MG208923	16S 27	OR918431		MG209048	ITS2 29	OR917405		
						Tur5-2		MG208924	16S 28	OR918432						
23.	43°44'26.18"N, 12°17'13.71"E	Italy, Tuscany, Sasso di Simone, Rifugio Casa del Re (Sestino, Arezzo)	G. Manganelli / 21.10.2017 / 4 (FGC 47484)	*M.cantiana*	CAN-2	Sim-1	COI 11	OR918547	16S 22	OR918433	H3 1	OR939930				
						Sim-2	COI 11	OR918548	16S 23	OR918434	H3 1	OR939931				
24.	45°11'59.85"N, 10°58'49.30"E	Italy, Venetum, Sorgà (Verona)	A. Hallgass / 09.2012 / 6 (FGC 42964)	*M.cantiana*	CAN-2	12FG-1		MG208925	16S 29	OR918435		MG209050	ITS2 30	OR917406		
						12FG-2		MG208928	16S 30	OR918436	H3 1	OR939932	ITS2 31	OR917407		
25.	48°15'25.50"N, 16°30'46.38"E	Austria, Breitenlee, abandoned railway station	M. Duda / 09.2015 / 3 (FGC 44020)	*M.cantiana*	CAN-3	Dud-2		MG208938	16S 31	OR918437		MG209056	ITS2 32	OR917408		
26.	43°46'11.79"N, 07°22'21.50"E	France, Alpes-Maritimes, Vallée de Peillon, Sainte Thècle	A. Hallgass / 24.10.2011/ 5 (FGC 40320)	*M.cemenelea*	CAN-4	3FG-1		MG208939	16S 32	OR918438		MG209058	ITS2 33	OR917409		
						3FG-2		MG208940	16S 32	OR918439		MG209059	ITS2 34	OR917410		

* Acronyms for collections: DCBC – the collection of the Department of Cell Biology, Adam Mickiewicz University, Poland; FGC – the Folco Giusti collection at Dipartimento di Scienze Fisiche, della Terra e dell’Ambiente, Università di Siena, Italy; MNHW – the Małgorzata Proćków collection at the Museum of Natural History, University of Wrocław, Poland.

### ﻿Material examined

The material examined originated from the populations listed in Table 1 with the following data: geographic coordinates, country and region, short description of collection site, name of collector, date, number of specimens studied and the collection where the material is stored (in brackets). The origin of the material used for comparison has been described in previous publications (Pieńkowska et al. 2015: appendix 1; Pieńkowska et al. 2018b, 2019a, 2019b, 2020, 2022: table 1).

### ﻿Morphological study

Sixty-six specimens of the six lineages of *M.cantiana* s.l. (CAN-1, CAN-2, CAN-3, CAN-4, CAN-5, and CAN-6) (Pieńkowska et al. 2018b, 2019a) and five specimens suitable for morphological analysis of the French populations were considered for shell variability (Table 1). Twelve shell variables were measured to the nearest 0.1 mm using ADOBE PHOTOSHOP 7.0.1 on digital images of standard apertural and umbilical views taken with a Canon EF 100 mm 1:2.8 L IS USM macro lens mounted on a Canon F6 camera (see also Pieńkowska et al. 2018b: fig. 1):

**AH** aperture height,

**AW** aperture width,

**LWfW** last whorl final width,

**LWmW** last whorl medial width,

**LWaH** height of adapical sector of last whorl,

**LWmH** height of medial sector of last whorl,

**PWH** penultimate whorl height,

**PWfW** penultimate whorl final width,

**PWmW** penultimate whorl medial width,

**SD** shell diameter,

**SH** shell height,

**UD** umbilicus diameter.

Sixty-four specimens of the six lineages of *M.cantiana* s.l. (CAN-1, CAN-2, CAN-3, CAN-4, CAN-5 and CAN-6) (Pieńkowska et al. 2018b, 2019a) and seven adult specimens of the French populations were analysed for anatomical variability (Table 1). Snail bodies were dissected under a light microscope (Wild M5A or Zeiss SteREO Lumar V12). Anatomical details were drawn using a Wild camera lucida. Abbreviations/acronyms are as follows (see also Pieńkowska et al. 2018b: fig. 2):

**Figure 2.**
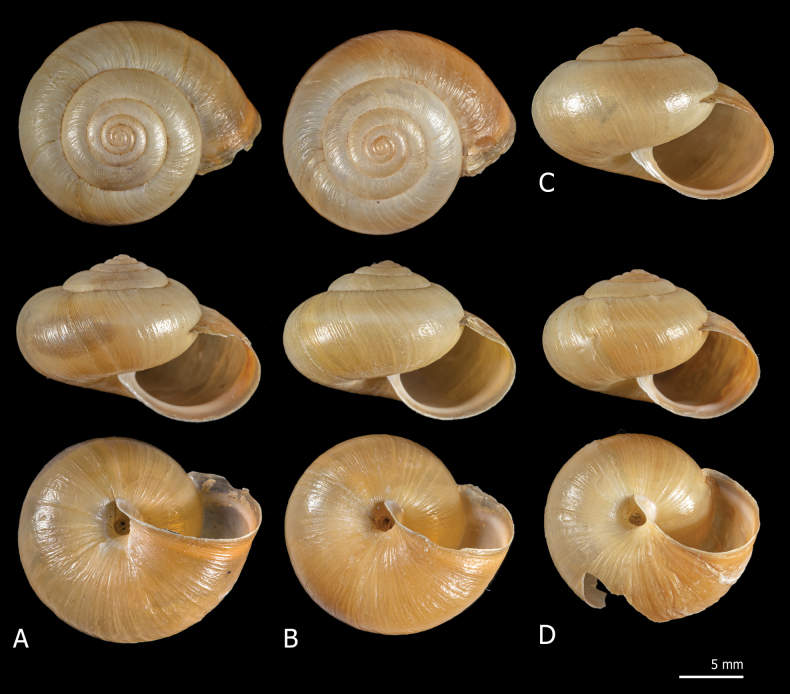
Shells of *Monachacantiana* from France. Specimen Esc1 from Oise, Escales-Saint-Pierre (**A**), specimen Ble1 from Pas-de-Calais, Blecquenecques n. Marquise (**B**), specimen Pie1 from Seine-Maritime, Pierrepont (**C**) and specimen Bet1 from Seine-Maritime, Béthencourt n. Grandcourt (**D**).

**BC** bursa copulatrix,

**BW** body wall,

**DBC** duct of bursa copulatrix,

**DG** digitiform glands,

**E** epiphallus (from base of flagellum to beginning of penial sheath),

**F** flagellum,

**FO** free oviduct,

**GA** genital atrium,

**GAR** genital atrium retractor,

**P** penis,

**PP** penial papilla,

**SOD** spermoviduct,

**V** vagina,

**VA** vaginal appendix (also known as appendicula),

**VAS** vaginal appendix sac,

**VD** vas deferens.

Six anatomical variables (DBC, E, F, P, V, VA) were measured using a calliper under a light microscope (0.01 mm) (Pieńkowska et al. 2018b: fig. 2).

Detailed methods of multivariate ordination by Principal Component Analysis (PCA) and Redundancy Analysis (RDA), performed on the original shell and genitalia matrices as well as on the Z-matrices (shape-related matrices), are described in our previous papers (Pieńkowska et al. 2018b, 2019a).

We used 95% confidence interval ellipses to evaluate the uncertainty of the estimate of the population mean (centroid) of the data sample. The function *ordiellipse* with standard errors in the package *vegan* (Oksanen et al. 2022) was used. Convex hulls (function *ordihull* in *vegan*) were used to visually enclose the individuals forming each clade as a measure of data spread. All analyses were performed with RStudio (R version 4.2.1; R Core Team 2021).

### ﻿Molecular study

Eighty-eight specimens representing 26 populations of the four lineages of *M.cantiana* s.l. (CAN-1, CAN-2, CAN-3, and CAN-4; Pieńkowska et al. 2018b, 2019a) were used for molecular analysis (Table 1). Molecular methods including DNA extraction, amplification and sequencing are described in our previous paper (Pieńkowska et al. 2018a).

Two mitochondrial and two nuclear gene fragments were analysed, namely cytochrome c oxidase subunit 1 (COI), 16S ribosomal DNA (16SrDNA), histone 3 (H3) and an internal transcribed spacer 2 of rDNA (ITS2) flanked by the 3’end of 5.8SrDNA and the 5’end of 28SrDNA, respectively. Sequences were edited by eye using BioEdit, v. 7.0.6 (Hall 1999; BioEdit 2017) and aligned using ClustalW, implemented in BioEdit (Thompson et al. 1994). Fragments of COI were amplified using two pairs of primers: F01/R04 (Dabert et al. 2010) or bcsmF1/bcsmR1 (Proćków et al. 2013). Fragments of 16SrDNA were amplified using 16Scs1/16Scs2 primers (Chiba 1999). Sequences containing the 3’end of 5.8SrDNA, complete sequence of ITS2 and 5’end of 28SrDNA were amplified using two sets of primers: LSU1/LSU3 (Wade and Mordan 2000) and NEWS2/ITS2-RIXO (Almeyda-Artigas et al. 2000). Products of the two PCR reactions were aligned and used to assemble single sequences. Fragments of H3 gene were amplified using the primers H3F and H3R (Colgan et al. 1998). The protein coding sequences were aligned according to the translated amino acid sequences. The ends of all sequences were trimmed. After trimming, the lengths of sequences were 615 bp for COI, 804–821 bp for 16SrDNA, 303 bp for H3, and 749–753 bp for ITS2 flanked by the 3’end of 5.8SrDNA and 5’end of 28SrDNA (including 45 bp 5.8SrDNA + 489–493 bp ITS2 + 215 bp 28SrDNA). The borders of ITS2 sequence were searched using ITS2-Database (http://its2.bioapps.biozentrum.uni-wuerzburg.de) (Eddy 1998; Koetschan et al. 2010). The sequences were collapsed to haplotypes using the programme ALTER (Alignment Transformation EnviRonment) (Glez-Peña et al. 2010). The following alignments were made for phylogenetic inference: 591 bp long for COI, 292 or 809 positions long for 16SrDNA, and 775 positions long for ITS2 flanked by the 3’end of 5.8SrDNA and 5’end of 28SrDNA. Finally, the sequences of COI, 16SrDNA, ITS2, and H3 were concatenated. Three sets of concatenated sequences were created: 1) COI16S of 1444 positions in length (615 COI + 829 16SrDNA); 2) H3ITS2 of 1054 positions in length (279 H3 + 775 ITS2 with flanks); 3) CS of 2498 positions in length (615 COI + 829 16SrDNA + 279 H3 + 775 ITS2 with flanks).

Estimates of genetic distances between the COI sequences obtained in this study and other sequences from GenBank were conducted with MEGA7 using the Kimura two-parameter model (K2P) (Kimura 1980). All positions containing gaps and missing data were eliminated. There were a total of 591 positions in the final dataset. The analysis involved 53 nucleotide sequences.

To infer the phylogenetic relationships the following programmes were used: MEGA7 (Hasegawa et al. 1985; Nei and Kumar 2000; Kumar et al. 2016), IQ-Tree (http://iqtree.cibiv.univie.ac.at/) (Trifinopoulos et al. 2016), RAxML v1.0.0 (Stamatakis 2014) and MrBayes 3.2.6 (Ronquist et al. 2012). For phylogenetic inference Neighbour-Joining, Maximum-Likelihood and Bayesian Inference methods were used.

For each alignment file, best nucleotide substitution models were specified according to the Bayesian Information Criterion (BIC) (see captions to figures). Phylogenetic analyses performed with IQ-Tree, RAxML and MrBayes for three sets of concatenated sequences were done dividing the data set into 2 or 4 partitions: (1) COI, (2) 16SrDNA or (1) COI, (2) 16SrDNA, (3) H3, (4) 5.8SrDNA + ITS2 + 28SrDNA. Best substitution models were inferred according to the Bayesian Information Criterion (BIC) for each of the partitions by MODELFINDER (Kalyaanamoorthy et al. 2017) implemented in IQ-TREE. Bayesian analysis were conducted with four Monte Carlo Markov chains running for 1 million generations, sampling every 100 generations (the first 25% of trees were discarded as ‘burn-in’).

The robustness of the NJ and ML trees generated by MEGA7 were assessed by bootstrap analysis with 1000 replicates (Felsenstein 1985). ML trees built by RAxML were tested by bootstrap analysis with 100 replicates. ML trees obtained with IQ-Tree were constructed under 1000 ultrafast bootstrap replicates (Minh et al. 2013). Finally, BI trees were supported by posterior probability (PP) values. Bootstrap support values from NJ and ML analysis as well as posterior probability (PP) values obtained on 50% majority rule consensus Bayesian tree were mapped onto the ML tree obtained by MEGA7. All the resulting trees were rooted with *Trochulushispidus* sequences obtained from GenBank.

## ﻿Results

### ﻿Morphological study: shell

Shells of French specimens of *M.cantiana* (Fig. 2A–D) are globose-subglobose in shape, variable in size and usually whitish or pale yellowish in colour, with slightly descending, roundish to oval aperture, similar to those of the other populations of the lineage CAN-1 (Pieńkowska et al. 2018b: figs 8–11).

**Figure 3.**
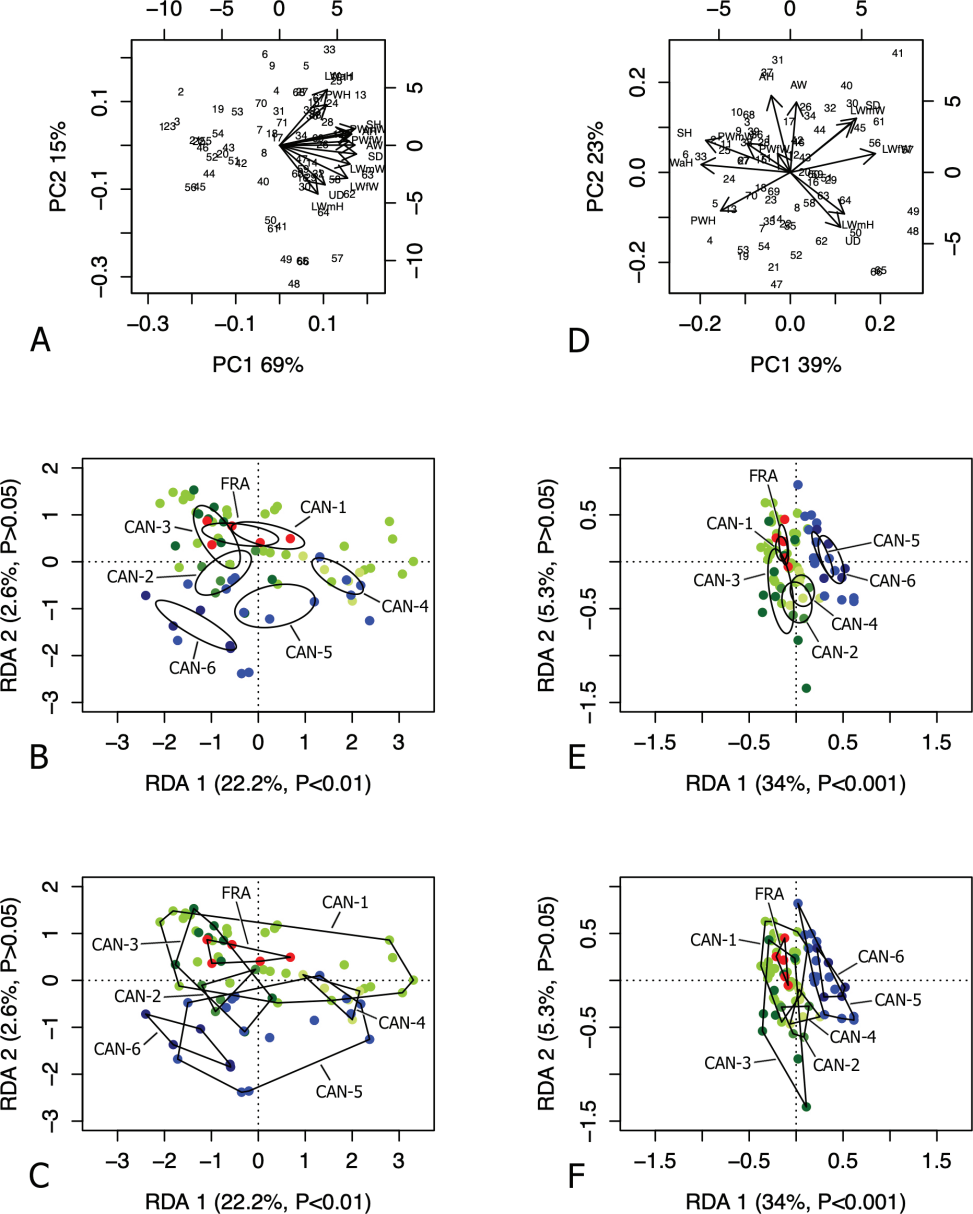
Analysis of French specimens with “lineage” constraint on the original matrix (**A–C**) and Z-matrix (shape-related) (**D–F**) of selected shell sections. Principal component analysis (PCA) (**A, D**) and redundancy analysis (RDA) with groups shown as ellipses representing 95% confidence intervals with standard errors (**B, E**) and as convex hull polygons (**C, F**).

RDA with French specimens and “lineage” constraint on the shape and size matrix (Fig. 3B, C) showed that RDA 1 (22.2%, P < 0.01) separated CAN-6 from CAN-4, with CAN-5 and the large group CAN-1, CAN-2, CAN-3, and FRA in intermediate position, as confirmed by 95% confidence interval ellipses (Fig. 3B). The convex hull measure of data spread showed considerable overlap of some clusters. In both cases, FRA specimens fell within CAN-1 variability (Fig. 3B). The preliminary classic PCA showed that size was the first major source of morphological variation, since PC1 (69%) was a positive combination of all variables (Fig. 3A). On the contrary, RDA 2 was not significant (p > 0.05) and accounted for little morphological variation (2.6%). PC2 (15%) mostly reflected a contrast between LWaH and PWH versus LWmH and UD.

RDA on the shape (Z) matrix (Fig. 3E, F) showed that RDA 1 (34%, P < 0.001) clearly separated CAN-5 and CAN-6 from the group CAN-1, CAN-2, CAN-3, CAN-4, and FRA, as confirmed by the 95% confidence interval ellipses (Fig. 3E) and the convex hulls (Fig. 3F). On the contrary, the RDA2 axis was not significant (P > 0.05), reflecting little morphological variation (5.3%). Shape-related PCA indicated that SH, LWaH and PWH vs LWmW, SD, LWfW, LWmH, and UD were the principal shape determinants on PC1, and AH and AW vs PWH, LWmH, and UD on PC2 (Fig. 3D).

### ﻿Morphological study: anatomy

**Figure 4.**
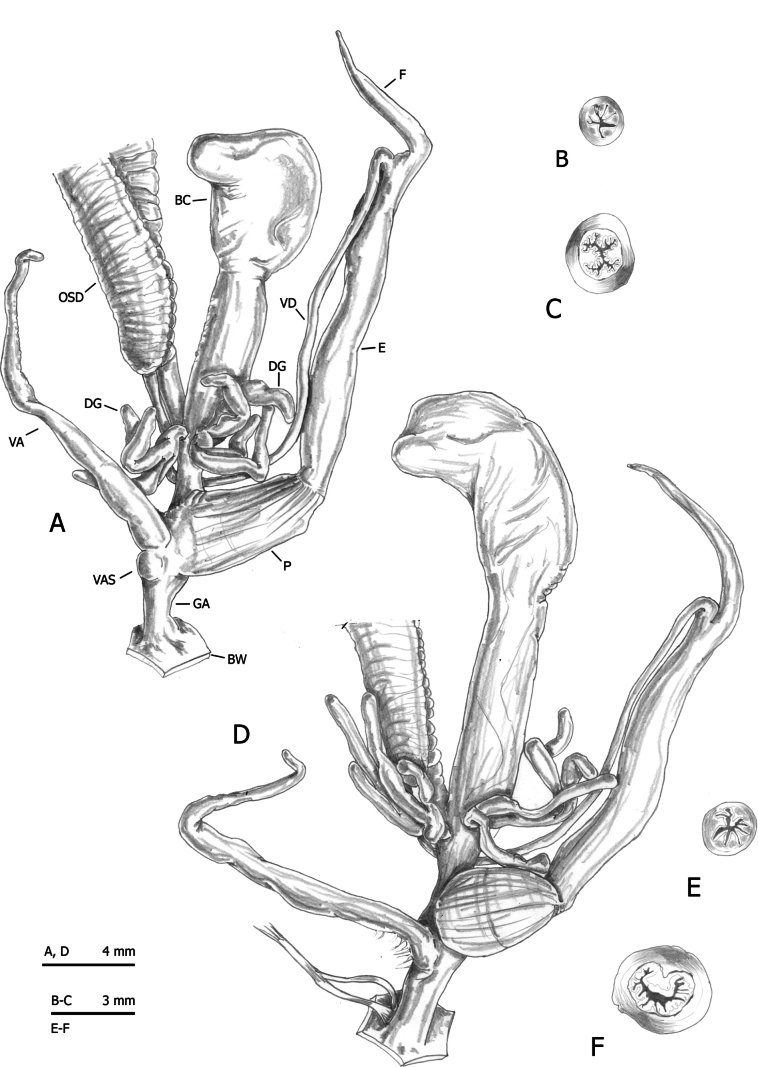
Distal genitalia of *Monachacantiana* from France. Specimen Bet1 from Seine-Maritime, Béthencourt n. Grandcourt (**A–C**) and specimen Ble1 from Pas-de-Calais, Blecquenecques n. Marquise (**D–F**). Distal genitalia (**A, D**), transverse sections of medial epiphallus (**B, E**) and apical penial papilla (**C, F**).

**Figure 5.**
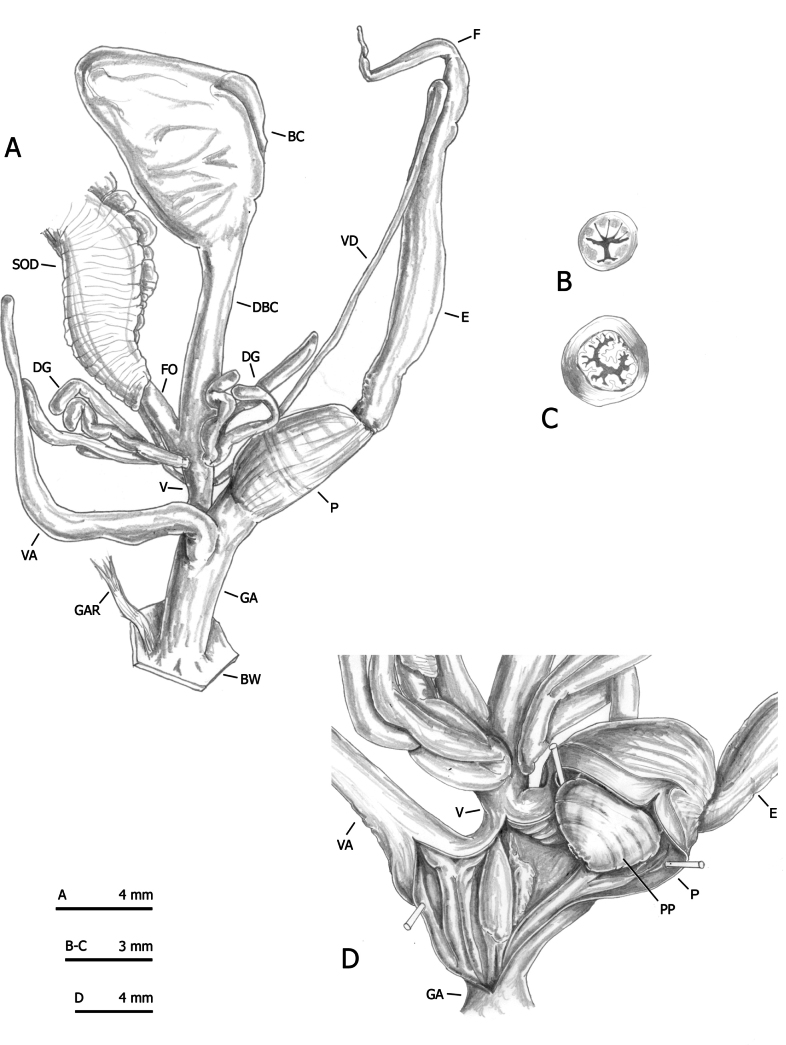
Distal genitalia genitalia of *Monachacantiana* from France. Specimen Esc1 from Oise, Escales-Saint-Pierre (**A–C**) and specimen Epa1 from Somme, Épagne-Épagnette, roadside (**D**). Distal genitalia (**A**), transverse sections of medial epiphallus (**B**), apical penial papilla (**C**) and internal structure of distal genitalia (**D**).

**Figure 6.**
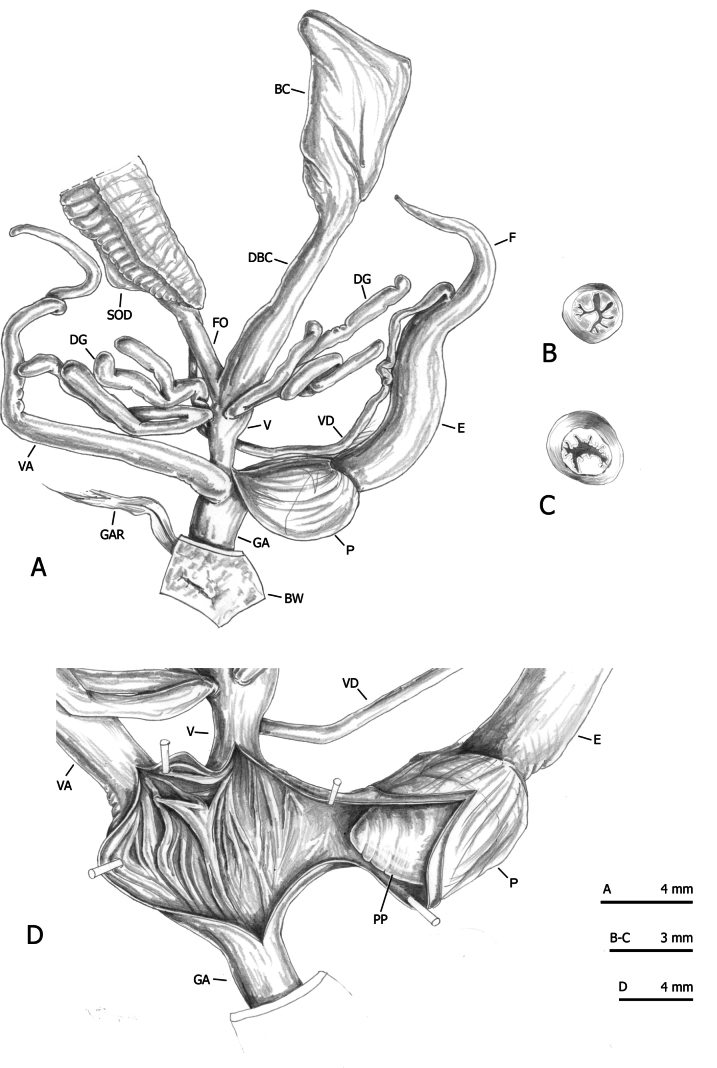
Distal genitalia of of *Monachacantiana* from France. Specimen Pie1 from Seine-Maritime, Pierrepont. Distal genitalia (**A**), transverse sections of medial epiphallus (**B**), apical penial papilla (**C**) and internal structure of distal genitalia (**D**).

French specimens of *M.cantiana* have distal genitalia (Figs 4–6) resembling the other populations assigned to CAN-1, which are in turn similar to those of the populations belonging to the CAN-2, CAN-3 and CAN-4 lineages (Pieńkowska et al. 2018b: figs 20–30).

**Figure 7.**
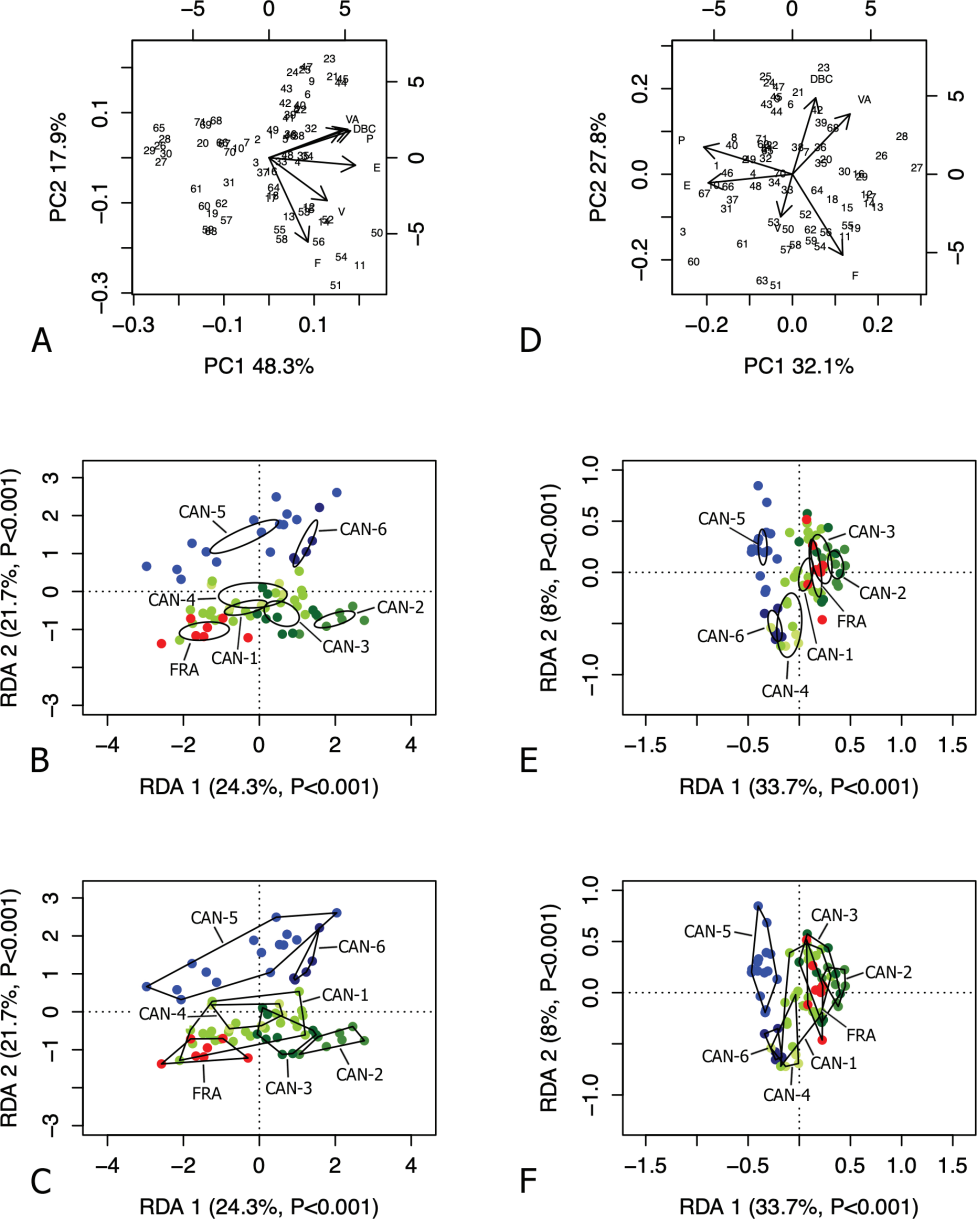
Analysis of French specimens with “lineage” constraint on the original matrix (**A–C**) and Z-matrix (shape-related) (**D–F**) of selected genital sections. Principal component analysis (PCA) (**A, D**) and redundancy analysis (RDA) with groups shown as ellipses representing 95% confidence intervals with standard errors (**B, E**) and as convex hull polygons (**C, F**).

RDA with French specimens and “lineage” constraint on the shape and size matrix (Fig. 7B, C) showed that RDA 1 (24.3%, P < 0.001) separated CAN-2 and CAN-6 from FRA and CAN-5, with CAN-1, CAN-3, CAN-4 in intermediate position, as confirmed by 95% confidence interval ellipses (Fig. 7B). The preliminary classic PCA showed that size was the first major source of morphological variation, since PC1 (48.3%) was a positive combination of all variables (Fig. 7A). On the other hand, RDA 2 (21.7%, P < 0.001) clearly separated the group CAN-1, CAN-2, CAN-3, CAN-4 and FRA from CAN-5 and CAN-6. PC2 (17.9%) reflected a contrast between P, VA and DBC vs F and V. Differences between clusters were confirmed visually by 95% confidence interval ellipses (Fig. 7B) and convex hulls (Fig. 7C).

RDA on the shape (Z) matrix (Figs 7E, F) showed that RDA 1 (33.7%, P < 0.001) separated the 95% confidence interval ellipses of CAN-5, CAN-6 and CAN-4 from the large group CAN-1, CAN-2, CAN-3, and FRA; RDA 2 (8%, P < 0.001) separated CAN-5 and the group CAN-1, CAN-2, CAN-3, FRA from CAN-6 and CAN-4 (Fig. 7E). Convex hulls showed some overlaps, especially in the data spread of CAN-1 (Fig. 7F). Shape-related PCA indicated that P and E vs VA and F were the two principal shape determinants on PC1 and DBC and VA vs V and F on PC2 (Fig. 7D).

### ﻿Molecular study

Although sequences of all the genes analysed (COI, 16SrDNA, H3, and ITS2 with 5.8SrDNA and 28SrDNA) were not obtained from all 88 specimens (Table 1), as a result of molecular analysis, 272 new sequences were deposited in GenBank. These were 56 new sequences of COI: OR918493–OR918548, 77 of 16SrDNA (long): OR918363–OR918439, 75 of H3: OR939858–OR939932 and 64 of ITS2 (with flanking fragments of 5.8SrDNA and 28SrDNA): OR917347–OR917410 (Table 1). Eleven haplotypes of the COI gene were identified (COI 1 – COI 11), 32 of 16SrDNA (16S 1 – 16S 32), 10 of H3 (H3 1 – H3 10), and 34 of ITS2 with flanking sequences (ITS2 1 – ITS2 34) (Table 1). These haplotypes were used for phylogenetic analysis based on single gene sequences and concatenated mitochondrial and nuclear gene data sets of sequences.

**Figure 8.**
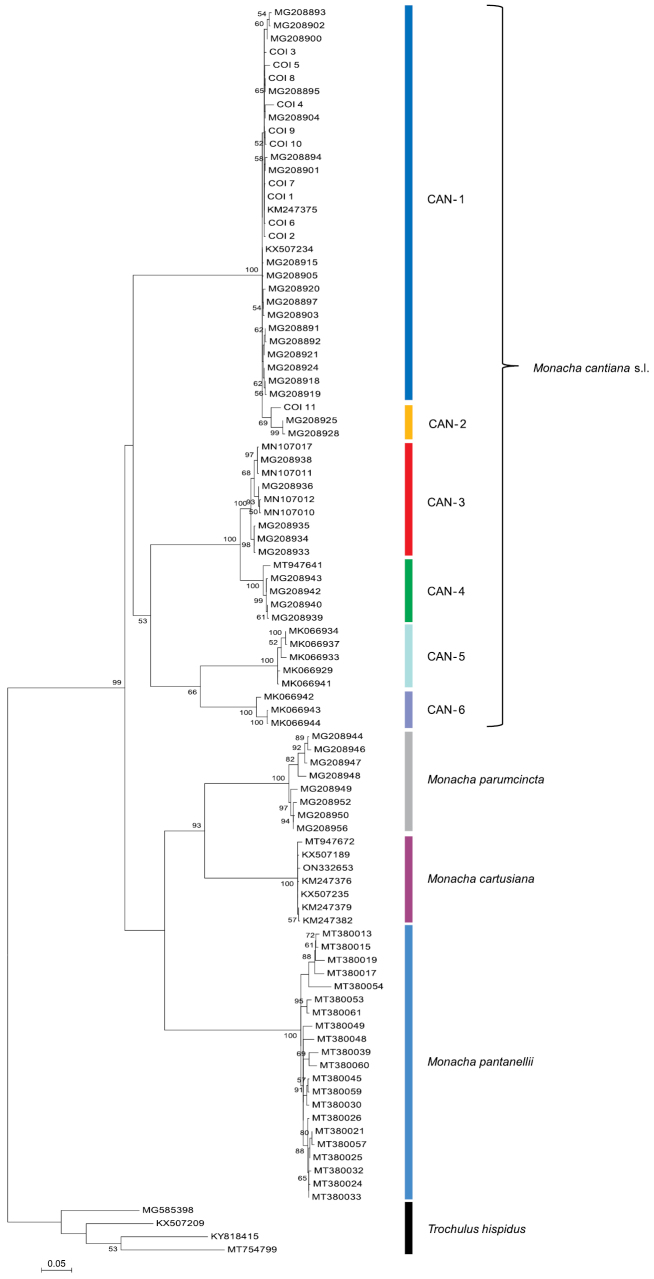
Maximum Likelihood (ML) tree of COI haplotypes of *Monachacantiana*. New COI sequences of *M.cantiana* (Table 1) were compared with COI sequences of *M.cantiana* s.l., *M.parumcincta*, *M.pantanellii* and *M.cartusiana* obtained from GenBank (Suppl. material 1). Sequences were cut to 591 bp. HKY+G+I was the best nucleotide substitution model according to the Bayesian Information Criterion (BIC). The tree was rooted with *Trochulushispidus* sequences obtained from GenBank (Suppl. material 1).

The phylogenetic analysis of COI sequences obtained from the specimens and comparative sequences derived from GenBank is shown in Fig. 8. The results are consistent with previously published findings (Pieńkowska et al. 2018b, 2019a, 2019b, 2020, 2022), distinguishing six lineages (CAN-1 – CAN-6) in *M.cantiana* s.l. that clustered separately from COI sequences of other species including *M.parumcincta*, *M.pantanellii* and *M.cartusiana*. The new COI sequences (haplotypes 1–10) from France, the Netherlands and England clustered in the CAN-1 lineage. Only the COI 11 haplotype obtained from two specimens of the Italian population from Sasso di Simone (population no. 23 in Table 1) grouped with the CAN-2 lineage.

K2P genetic distances (Table 2) showed small genetic differentiation between COI sequences of particular CAN-1 populations (infra-populational distances ranged from 0.2% in Dutch populations to 1.1% in French populations). The K2P distances between these populations were also small (in the range 0.5–1.2%). The K2P distances between French, Dutch, English and Italian populations of CAN-1 and CAN-2 were also small (in the range 3.5–4.1%) while the distance separating the CAN-1 populations from the CAN-3 and CAN-4 populations was much larger (in the range 18.0–18.8%). In turn, the distance separating the CAN-3 and CAN-4 populations was 5.6–6.1%.

**Table 2.** K2P genetic distances between COI sequences of the populations analysed.

		1	2	3	4	5	6	7	8
*M.cantiana* CAN-1 of French populations	**1**	**1.1**							
*M.cantiana* CAN-1 of Dutch populations	**2**	0.7	**0.2**						
*M.cantiana* CAN-1 of English populations	**3**	0.9	0.5	**0.7**					
*M.cantiana* CAN-1 of Italian populations	**4**	1.2	0.8	0.9	**0.6**				
*M.cantiana* CAN-2 of Italian populations	**5**	4.1	3.7	3.8	3.5	**2.4**			
*M.cantiana* s.l. CAN-3 of Italian populations	**6**	18.7	18.6	18.6	18.5	18.3	**1.0**		
*M.cantiana* s.l. CAN-3 of Austrian populations	**7**	18.8	18.7	18.7	18.7	18.5	1.5	**1.0**	
*M.cantiana* s.l. CAN-4 (*M.cemenelea*) of French populations	**8**	18.3	18.2	18.1	18.0	18.6	5.6	6.1	**0.9**

Results similar to those of COI analysis were obtained for other single gene analyses (Suppl. materials 8, 9 for 16SrDNA, Suppl. material 10 for the ITS2 gene with flanking 5.8S and 28S gene fragments). Note that the newly obtained 16SrDNA sequences in Suppl. material 8 were trimmed to 292 positions in alignment length because GenBank lacks the reference long 16SrDNA sequences of the 809 positions used to construct the tree in Suppl. material 9. Analysis of newly obtained longer sequences (i.e. ITS2 flanked by 5.8SrDNA and 28SrDNA gene fragments) (ITS2 1 – ITS2 34 haplotypes) and the only comparable sequence of Neiber and Hausdorf (2017) showed that this gene did not differentiate the CAN-1, CAN-2 and CAN-3 lineages. Similar results were obtained previously using ITS2 gene sequences without flanking fragments of 5.8SrDNA and 28SrDNA (Pieńkowska et al. 2018b: fig. 64). Only in the case of sequences assigned to the CAN-4 lineage were they distinct from CAN-1, CAN-2 and CAN-3, as shown in Pieńkowska et al. (2018b: fig. 64).

**Figure 9.**
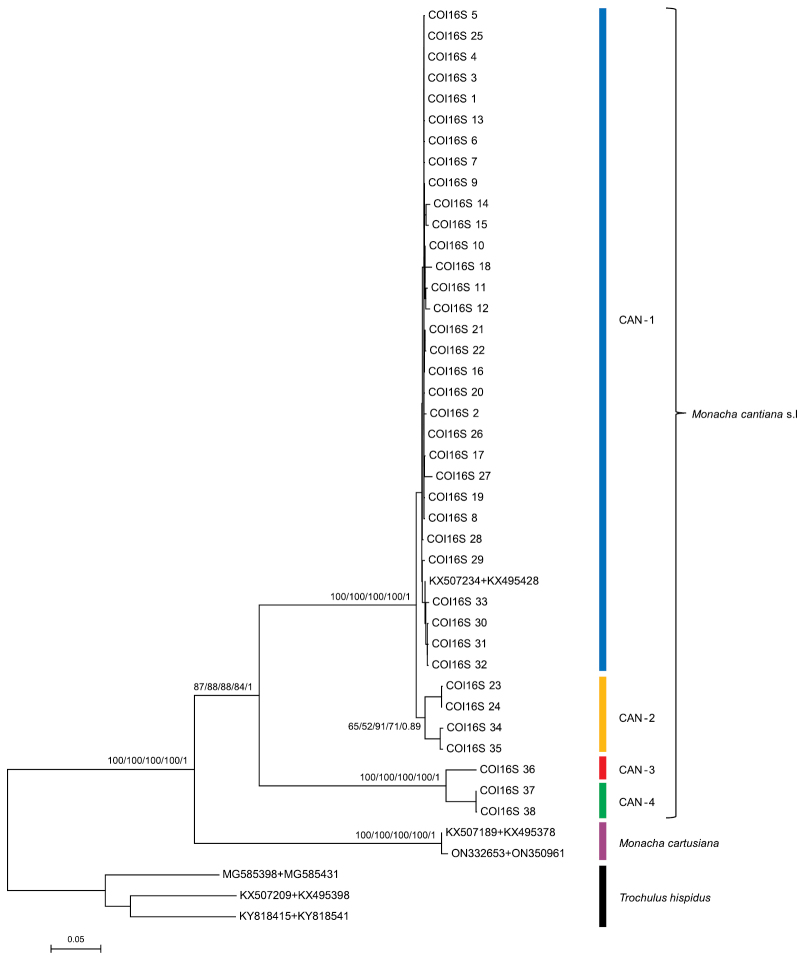
Maximum Likelihood (ML) tree of concatenated COI and 16SrDNA haplotypes of *Monachacantiana*. New COI and 16SrDNA sequences of *M.cantiana* (Table 1, Suppl. material 5) were compared with concatenated COI and 16SrDNA sequences of *M.cantiana* s.l. and *M.cartusiana* obtained from GenBank (Suppl. materials 1, 2, 5). Length of sequences was 1444 positions (615 of COI + 829 of 16SrDNA). The Bayesian Information Criterion (BIC) specified T92+G+I the best nucleotide substitution model in MEGA7, or HKY+F+G4 for COI and TIM2+F+G4 for 16SrDNA partition in IQ-Tree, RAxML and MrBayes. Numbers next to main branches indicate (left to right): bootstrap supports above 50% calculated by NJ-MEGA7 (Saitou and Nei 1987), ML-MEGA7 (Kumar et al. 2016), IQ-Tree (Trifinopoulos et al. 2016), RAxML (Stamatakis 2014), and posterior probabilities by BI (Ronquist et al. 2012). The tree was rooted with *Trochulushispidus* concatenated sequences obtained from GenBank (Suppl. material 5).

**Figure 10.**
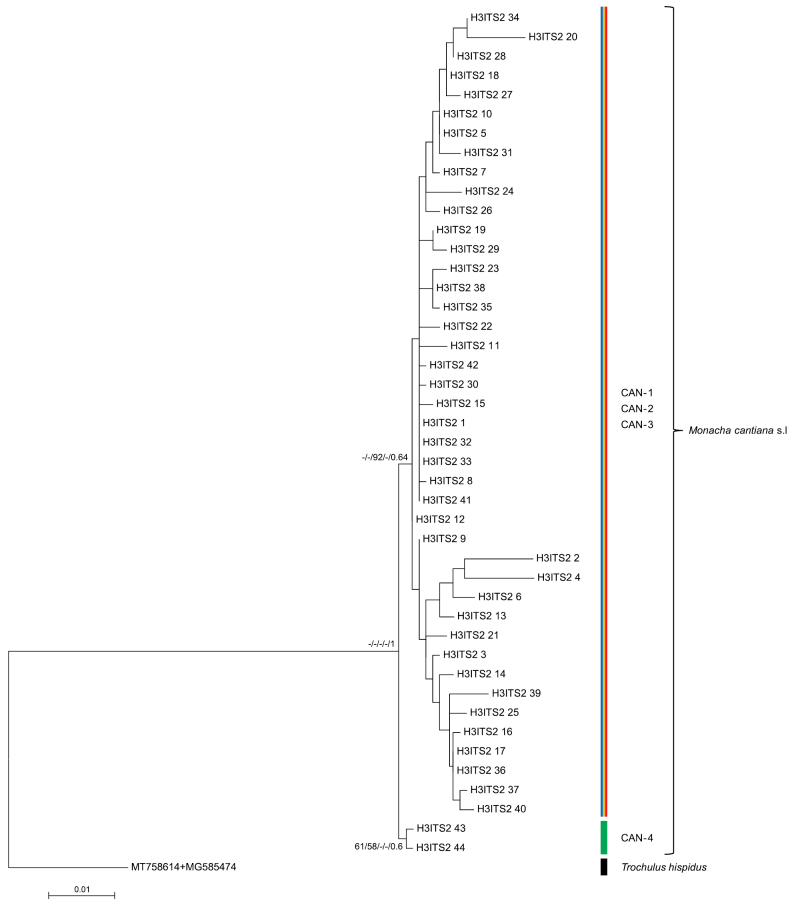
Maximum Likelihood (ML) tree of concatenated H3 and ITS2 (flanked with 5.8S and 28SrDNA) haplotypes of *Monachacantiana*. New H3 and ITS2 sequences of *M.cantiana* (Table 1) were compared with concatenated H3 and ITS2 sequences of *M.cantiana* s.l. obtained from GenBank (Suppl. materials 3, 4). Length of sequences was 1054 positions (279 of H3 + 775 of ITS2). The Bayesian Information Criterion (BIC) specified T92+G+I the best nucleotide substitution model in MEGA7, or K2P+I for H3 and K3P+I for ITS2 partition in IQ-Tree, RAxML, and MrBayes. Numbers next to main branches indicate (left to right): bootstrap supports above 50% calculated by NJ-MEGA7 (Saitou and Nei 1987), ML-MEGA7 (Kumar et al. 2016), IQ-Tree (Trifinopoulos et al. 2016), RAxML (Stamatakis 2014) and posterior probabilities by BI (Ronquist et al. 2012). The tree was rooted with *Trochulushispidus* concatenated sequences obtained from GenBank (Suppl. material 6).

**Figure 11.**
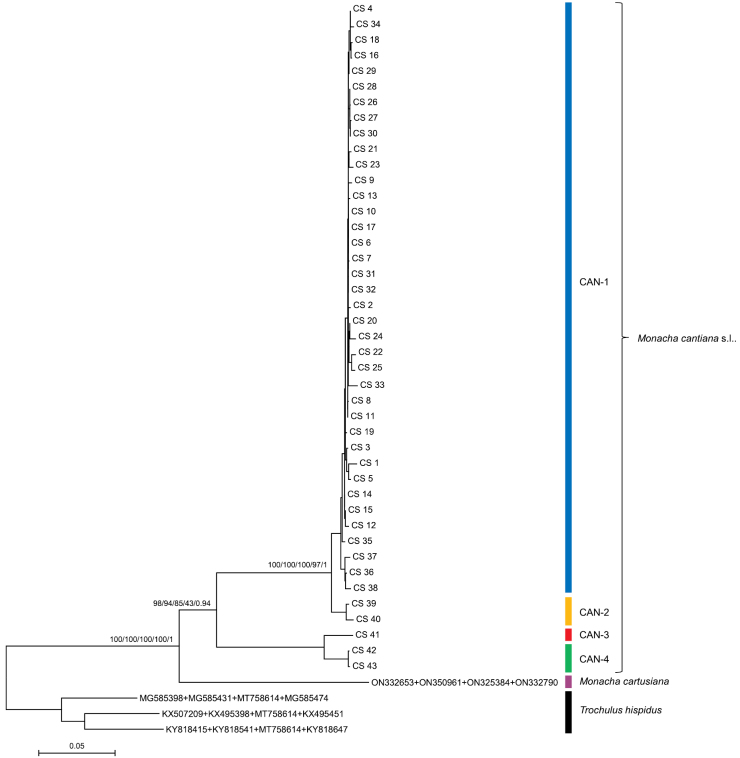
Maximum Likelihood (ML) tree of concatenated COI, 16SrDNA, H3, and ITS2 (flanked with 5.8S and 28SrDNA) haplotypes of *Monachacantiana*. COI, 16SrDNA, H3, and ITS2 sequences of *M.cantiana* were compared with sequences of *M.cantiana* s.l. and *M.cartusiana* obtained from GenBank (Suppl. materials 1–4, 7). Length of sequences was 2498 positions (615 of COI, 829 of 16SrDNA, 279 of H3, and 775 of ITS2). Bayesian Information Criterion (BIC) specified GTR+G+I the best nucleotide substitution model in MEGA7, or HKY+F+G4 for COI, TIM2+F+I for 16SrDNA, TIM3e+I+G4 for H3, and K3P+I+G4 for ITS2 partition in IQ-Tree, RAxML, and MrBayes. Numbers next to main branches indicate (left to right): bootstrap support above 50% calculated by NJ-MEGA7 (Saitou and Nei 1987), ML-MEGA7 (Kumar et al. 2016), IQ-Tree (Trifinopoulos et al. 2016), RAxML (Stamatakis 2014), and posterior probabilities by BI (Ronquist et al. 2012). The tree was rooted with *Trochulushispidus* concatenated sequences obtained from GenBank (Suppl. material 7).

The phylogenetic tree for concatenated sequences were similar in ML analyses obtained with different software. The tree for mitochondrial gene sequences (COI+16SrDNA) in Fig. 9 shows that the sequences obtained from specimens of the French, Dutch, and English populations (see also Suppl. material 5) grouped with the reference sequences for CAN-1. In a tree of concatenated nuclear genes (Fig. 10: H3+ITS2 with flanks), the sequences from the French populations grouped with CAN-1, CAN-2, and CAN-3 lineages, only sequences of the CAN-4 lineage being distinguished. However, note that the bootstrap and posterior probability values weakly supported the results of the concatenated H3+ITS2 gene sequences. The tree for the concatenated sequences of all the genes analysed in this paper (Fig. 11, see also Suppl. material 7) showed that concatenated sequences CS 1–CS 25 from northern French populations clustered together with CS 26–CS 34 and CS 35–CS 38 sequences obtained from English and Italian specimens, respectively. They all belonged to the CAN-1 lineage. The CAN-1, CAN-2, CAN-3, and CAN-4 lineages grouped separately.

## ﻿Discussion

At a first glance, the shells and genitalia of the French specimens do not differ from those of the other populations assigned to CAN-1, which in turn are similar to those of the populations of the CAN-2, CAN-3 and CAN-4 lineages (see Pieńkowska et al. 2018b). This was fully confirmed by RDA and PCA: the French specimens fell entirely in CAN-1 on the basis of shell characters (Fig. 3C, F), and almost entirely, based on anatomical characters (Fig. 7C, F).

The results of molecular analysis were consistent with those of morphological analysis (shell and genital structure). Both showed that the populations from northern France should be assigned to the CAN-1 lineage. In this sense, the molecular results complement the conclusions of Brulé and Bichain (2019). Consequently, their results corroborate the results of four previous papers on *M.cantiana* lineages and their phylogeography (Pieńkowska et al. 2018b, 2019a, 2019b, 2020).

Prior suggestions that *M.cantiana* was introduced into England in historical times (Kerney 1970, 1999; Evans 1972; Pieńkowska et al. 2018b) appear to be correct. This allows us to speculate that the Roman conquests also spread *M.cantiana* in northern France (as well as in the area of modern-day Holland). The slightly greater genetic diversity of French populations compared to the English ones (expressed as slightly larger K2P distances) indicates that *M.cantiana* reached northern France earlier than England. Simultaneously, the occurrence of the CAN-2 and CAN-3 lineages in Italy implies that *M.cantiana* populations diversified for longer in this area. Nevertheless, further analysis of *M.cantiana*, especially specimens from northern Italy, is needed to determine the relationships between the CAN-1/CAN-2 and CAN-3/CAN-4 lineages. Until these results are available, we refrain from proposing any nomenclatural taxonomic framework for these lineages.

## ﻿Additional information

### Author ORCIDs

Joanna R. Pieńkowska https://orcid.org/0000-0003-0372-121X

Giuseppe Manganelli https://orcid.org/0000-0002-8453-280X

Małgorzata Proćków https://orcid.org/0000-0003-2240-7306

Debora Barbato https://orcid.org/0000-0003-1105-1711

Katarzyna Sosnowska http://orcid.org/0000-0002-7506-4231

Folco Giusti https://orcid.org/0000-0001-8722-4653

Andrzej Lesicki https://orcid.org/0000-0002-1924-1934

### Data availability

All of the data that support the findings of this study are available in the main text or Supplementary Information.

## Supplementary materials

Supplementary material 1 COI sequences from GenBank used for molecular analysis comparisons (haplotypes in bold) Data type docx https://binary.pensoft.net/file/1031126This dataset is made available under the Open Database License (http://opendatacommons.org/licenses/odbl/1.0/). The Open Database License (ODbL) is a license agreement intended to allow users to freely share, modify, and use this Dataset while maintaining this same freedom for others, provided that the original source and author(s) are credited.Joanna R. Pieńkowska, Giuseppe Manganelli, Małgorzata Proćków, Debora Barbato, Katarzyna Sosnowska, Folco Giusti, Andrzej Lesicki

Supplementary material 2 16SrDNA sequences from GenBank used for molecular analysis comparisons (haplotypes in bold) Data type docx https://binary.pensoft.net/file/1031127This dataset is made available under the Open Database License (http://opendatacommons.org/licenses/odbl/1.0/). The Open Database License (ODbL) is a license agreement intended to allow users to freely share, modify, and use this Dataset while maintaining this same freedom for others, provided that the original source and author(s) are credited.Joanna R. Pieńkowska, Giuseppe Manganelli, Małgorzata Proćków, Debora Barbato, Katarzyna Sosnowska, Folco Giusti, Andrzej Lesicki

Supplementary material 3 H3 sequences from GenBank used for molecular analysis comparisons (haplotypes in bold) Data type docx https://binary.pensoft.net/file/1031128This dataset is made available under the Open Database License (http://opendatacommons.org/licenses/odbl/1.0/). The Open Database License (ODbL) is a license agreement intended to allow users to freely share, modify, and use this Dataset while maintaining this same freedom for others, provided that the original source and author(s) are credited.Joanna R. Pieńkowska, Giuseppe Manganelli, Małgorzata Proćków, Debora Barbato, Katarzyna Sosnowska, Folco Giusti, Andrzej Lesicki

Supplementary material 4 ITS2 sequences from GenBank used for molecular analysis comparisons (haplotypes in bold) Data type docx https://binary.pensoft.net/file/1031129This dataset is made available under the Open Database License (http://opendatacommons.org/licenses/odbl/1.0/). The Open Database License (ODbL) is a license agreement intended to allow users to freely share, modify, and use this Dataset while maintaining this same freedom for others, provided that the original source and author(s) are credited.Joanna R. Pieńkowska, Giuseppe Manganelli, Małgorzata Proćków, Debora Barbato, Katarzyna Sosnowska, Folco Giusti, Andrzej Lesicki

Supplementary material 5 Concatenated sequences of COI+16SrDNA used in NJ/ML-MEGA7/IQ Tree/RAxML/BI analysis (Fig. 9) Data type docx Explanation note COI sequences were 615 bp in length. Long 16SrDNA sequences were cut to 829 positions (the alignment of concatenated sequences COI and long 16SrDNA was then 1444 positions in length). https://binary.pensoft.net/file/1031130This dataset is made available under the Open Database License (http://opendatacommons.org/licenses/odbl/1.0/). The Open Database License (ODbL) is a license agreement intended to allow users to freely share, modify, and use this Dataset while maintaining this same freedom for others, provided that the original source and author(s) are credited.Joanna R. Pieńkowska, Giuseppe Manganelli, Małgorzata Proćków, Debora Barbato, Katarzyna Sosnowska, Folco Giusti, Andrzej Lesicki

Supplementary material 6 Concatenated sequences of H3 + [(5.8SrDNA)+ITS2+(28SrDNA)] used in NJ/ML-MEGA7/IQ Tree/RAxML/BI analysis (Fig. 10) Data type docx Explanation note H3 sequences were cut to 279 bp, 5.8SrDNA+ITS2+28SrDNA sequences were 775 positions in length (the alignment of concatenated sequences H3 + [(5.8SrDNA)+ITS2+(28SrDNA)] was therefore 1054 positions). https://binary.pensoft.net/file/1031131This dataset is made available under the Open Database License (http://opendatacommons.org/licenses/odbl/1.0/). The Open Database License (ODbL) is a license agreement intended to allow users to freely share, modify, and use this Dataset while maintaining this same freedom for others, provided that the original source and author(s) are credited.Joanna R. Pieńkowska, Giuseppe Manganelli, Małgorzata Proćków, Debora Barbato, Katarzyna Sosnowska, Folco Giusti, Andrzej Lesicki

Supplementary material 7 Concatenated sequences of COI + 16SrDNA long + H3 + [(5.8SrDNA)+ITS2+(28SrDNA)] used in NJ/ML-MEGA7/ML-IQ Tree/RAxML/BI analysis (Fig. 11) Data type docx Explanation note The lengths of the particular sequences were COI 615 bp, 16SrDNA – 829 bp, H3 – 279 bp, 5.8SrDNA+ITS2+28SrDNA – 775 bp (the alignment of concatenated sequences COI + 16SrDNA long + H3 + [(5.8SrDNA)+ITS2+(28SrDNA)] was therefore 2498 positions). https://binary.pensoft.net/file/1031132This dataset is made available under the Open Database License (http://opendatacommons.org/licenses/odbl/1.0/). The Open Database License (ODbL) is a license agreement intended to allow users to freely share, modify, and use this Dataset while maintaining this same freedom for others, provided that the original source and author(s) are credited.Joanna R. Pieńkowska, Giuseppe Manganelli, Małgorzata Proćków, Debora Barbato, Katarzyna Sosnowska, Folco Giusti, Andrzej Lesicki

Supplementary material 8 Maximum Likelihood (ML) tree of 16SrDNA haplotypes of *Monachacantiana* Data type eps Explanation note New 16SrDNA sequences of *M.cantiana* (Table 1) were compared with 16SrDNA sequences of *M.cantiana* s.l., *M.parumcincta*, *M.pantanellii* and *M.cartusiana* from GenBank (Suppl. material 2). Sequences were cut to 292 positions. GTR+G+I (Nei and Kumar 2000; Kumar et al. 2016) was the best nucleotide substitution model according to the Bayesian Information Criterion (BIC). Numbers next to branches indicate bootstrap support above 50% calculated by ML-MEGA7 (Kumar et al. 2016) on 1000 replicates (Felsenstein 1985). The tree was rooted with *Trochulushispidus* sequences from GenBank (Suppl. material 2). https://binary.pensoft.net/file/1031133This dataset is made available under the Open Database License (http://opendatacommons.org/licenses/odbl/1.0/). The Open Database License (ODbL) is a license agreement intended to allow users to freely share, modify, and use this Dataset while maintaining this same freedom for others, provided that the original source and author(s) are credited.Joanna R. Pieńkowska, Giuseppe Manganelli, Małgorzata Proćków, Debora Barbato, Katarzyna Sosnowska, Folco Giusti, Andrzej Lesicki

Supplementary material 9 Maximum Likelihood (ML) tree of 16SrDNA haplotypes of *Monachacantiana* Data type eps Explanation note New 16SrDNA sequences of *M.cantiana* (Table 1) were compared with 16SrDNA sequences of *M.cantiana* s.l. and *M.cartusiana* from GenBank (Suppl. material 2). Sequences were cut to 809 positions. T92+G (Tamura 1992; Kumar et al. 2016) was the best nucleotide substitution model according to the Bayesian Information Criterion (BIC). Numbers next to branches indicate bootstrap support above 50% calculated by ML-MEGA7 (Kumar et al. 2016) on 1000 replicates (Felsenstein 1985). The tree was rooted with *Trochulushispidus* sequences from GenBank (Suppl. material 2). https://binary.pensoft.net/file/1031134This dataset is made available under the Open Database License (http://opendatacommons.org/licenses/odbl/1.0/). The Open Database License (ODbL) is a license agreement intended to allow users to freely share, modify, and use this Dataset while maintaining this same freedom for others, provided that the original source and author(s) are credited.Joanna R. Pieńkowska, Giuseppe Manganelli, Małgorzata Proćków, Debora Barbato, Katarzyna Sosnowska, Folco Giusti, Andrzej Lesicki

Supplementary material 10 Maximum Likelihood (ML) tree of ITS2 (flanked with 5.8S and 28SrDNA) haplotypes of *Monachacantiana* Data type eps Explanation note New ITS2 sequences of *M.cantiana* (Table 1) were compared with ITS2 sequences of *M.cantiana* s.l. and *M.cartusiana* from GenBank (Suppl. material 3). Sequences of specimens representing CAN-2 and CAN-3 lineages are shown. Sequences were cut to 775 positions. JC+G (Jukes and Cantor 1969; Kumar et al. 2016) was the best nucleotide substitution model according to the Bayesian Information Criterion (BIC). Numbers next to branches indicate bootstrap support above 50% calculated by ML-MEGA7 (Kumar et al. 2016) on 1000 replicates (Felsenstein 1985). The tree was rooted with *Trochulushispidus* sequences from GenBank (Suppl. material 4). https://binary.pensoft.net/file/1031135This dataset is made available under the Open Database License (http://opendatacommons.org/licenses/odbl/1.0/). The Open Database License (ODbL) is a license agreement intended to allow users to freely share, modify, and use this Dataset while maintaining this same freedom for others, provided that the original source and author(s) are credited.Joanna R. Pieńkowska, Giuseppe Manganelli, Małgorzata Proćków, Debora Barbato, Katarzyna Sosnowska, Folco Giusti, Andrzej Lesicki
