# Supplementary material for: ﻿Next step in Monachacantiana (Montagu, 1803) phylogeography: northern French and Dutch populations (Eupulmonata, Stylommatophora, Hygromiidae)
Source: Zookeys. 2024 Apr 23;1198:55–86. doi: 10.3897/zookeys.1198.119738 (PMC11061557; doi:10.3897/zookeys.1198.119738)
Supplement: Supplementary material 1 — COI sequences from GenBank used for molecular analysis comparisons (haplotypes in bold) [file zookeys-1198-055_article-119738__-s001.docx]

**Table S1**. COI sequences obtained from GenBank used for molecular analysis comparisons (haplotypes in bold)

| **species** | **COI** | **references** |
| --- | --- | --- |
| *Monacha cantiana* CAN-1 Spanish populations | **KX507234** | Neiber & Hausdorf (2017) KX |
| *Monacha cantiana* CAN-1 UK populations | **KM247375** (= MG208883-MG208890)  **MG208891**, **MG208892**, **MG208893**, **MG208894**, **MG208895** (= MG208896), **MG208897** (= MG208898-MG208899), **MG208900**, **MG208901**, **MG208902**, **MG208903**, **MG208904** | Pieńkowska et al. (2015) KM, Pieńkowska et al. (2018b) MG |
| *Monacha cantiana* CAN-1 Italian populations | **MG208905** (= MG208906-MG208909), MG208910-MG208914 (= MG208891), **MG208915** (= MG208916-MG208917), **MG208918**, **MG208919**, **MG208920**, **MG208921** (= MG208922-MG208923), **MG208924** | Pieńkowska et al. (2018b) MG |
| *Monacha cantiana* CAN-2 Italian populations | **MG208925** (= MG208926-MG208927, OP100313), **MG208928** (= MG208929-MG208932, OP100312, MN107013-MN107014) | Pieńkowska et al. (2018b) MG, Čejka et al. (2022) OP |
| *Monacha cantiana* s.l. CAN-3 Italian populations | **MG208933**, **MG208934**, **MG208935** | Pieńkowska et al. (2018b) MG |
|  | **MG208936** (= MG208937, HQ204502, KF596907, MN107013-MN107014, OP100311), **MG208938** (= MN107015-MN107016, MN107018-MN107019) | Duda et al. (2011) & Kruckenhauser et al. (2014) HQ, Cadahia et al. (2014) KF, Pieńkowska et al. (2018b) MG, Pieńkowska et al. (2019b) MN, Čejka et al. (2022) OP |
|  | **MN107010**, **MN107011**, **MN107012**, **MN107017** | Pieńkowska et al. (2019b) |
| *Monacha cantiana* s.l. CAN-4  = *Monacha cemenelea* French populations | **MG208939**, **MG208940** (= MG208941), **MG208942**, **MG208943** | Pieńkowska et al. (2018b) MG |
|  | **MT947641** | Čejka et al. (2020) MT |
| *Monacha cantiana* s.l. CAN-5 Italian populations | **MK066929** (= MK066930-MK066932, MK066938-MK0669940), **MK066933**, **MK066934** (= MK066935-MK066936), **MK066937**, **MK066941** | Pieńkowska et al. (2019a) MK |
| *Monacha cantiana* s.l. CAN-6 Italian populations | **MK066942**, **MK066943**, **MK066944** (= MK066945-MK066946) | Pieńkowska et al. (2019a) MK |
| *Monacha pantanellii* Italian populations | **MT380011** (= MT380012), **MT380013**, **MT380014**, **MT380015**, **MT380016**, **MT380017** (= MT380018), **MT380019**, **MT380021**, **MT380022, MT380023**, **MT3800224**, **MT3800225**, **MT380026**, **MT380027** (= MT380028-MT380029, MT380034- MT380037), **MT380030**, **MT380031**, **MT380032** (= MT380052), **MT380033**, **MT380038** (= MT380050), **MT380039**, **MT380040** (= MT380041- MT380042), **MT380043, MT380044**, **MT380045** (= MT380046-MT38047), **MT380048**, **MT380049**, **MT380053**, **MT380054**, **MT380057** (= MT380058), **MT380059**, **MT380060**, **MT380061** (= MT380062-MT380063) | Pieńkowska et al. (2020) MT |
| *Monacha parumcinta* Italian populations | **MG208944** (= MG208945), **MG208946**, **MG208947**, **MG208948**, **MG208949**, **MG208950** (= MG208951), **MG208952** (= MG208953-MG208955), **MG208956** (= MG208957-MG208959) | Pieńkowska et al. (2018b) MG |
| *Monacha cartusiana* Hungarian population | **KM247376** | Pieńkowska et al. (2015) KM |
| *Monacha cartusiana* Polish population | **KM247379** | Pieńkowska et al. (2015) KM |
| *Monacha cartusiana* Czech population | **KM247382** (= MT947671) | Pieńkowska et al. (2015) KM, Čejka et al. (2020) MT |
|  | MT947672 | Čejka et al. (2020) MT |
| *Monacha cartusiana* Italian population | **KX507189** (= KM247389) | Neiber & Hausdorf (2017) KX, Pieńkowska et al. (2015) KM |
| *Monacha cartusiana* Spanish population | **KX507235** (= MT947646) | Neiber & Hausdorf (2017) KX, Čejka et al. (2020) MT |
| *Monacha cartusiana* French populations | **MT947672** | Čejka et al. (2020) MT |
|  | **ON332653** | Pieńkowska et al. (2022) ON |
| *Trochulus hispidus* | **KX507209**, **KY818415**, **MG585398**, **MT754799** | Neiber & Hausdorf (2017) KX, Neiber et al. (2017) KY, Caro et al. (2019) MG, Proćków et al. (2021) MT |
