# Supplementary material for: ﻿Next step in Monachacantiana (Montagu, 1803) phylogeography: northern French and Dutch populations (Eupulmonata, Stylommatophora, Hygromiidae)
Source: Zookeys. 2024 Apr 23;1198:55–86. doi: 10.3897/zookeys.1198.119738 (PMC11061557; doi:10.3897/zookeys.1198.119738)
Supplement: Supplementary material 2 — 16SrDNA sequences from GenBank used for molecular analysis comparisons (haplotypes in bold) [file zookeys-1198-055_article-119738__-s002.docx]

**Table S2**. 16SrDNA sequences obtained from GenBank used for molecular analysis comparisons (haplotypes in bold)

| **species** | **16SrDNA short ***  **16SrDNA long **** | **references** |
| --- | --- | --- |
| *Monacha cantiana* CAN-1 Spanish populations | **KX495428**** | Neiber & Hausdorf (2017) KX |
|  | **KJ458539*** | Razkin et al. (2015) KJ |
| *Monacha cantiana* CAN-1 UK populations | **KM247390*** (= MG208960-MG208974) | Pieńkowska et al. (2015) KM, Pieńkowska et al. (2018b) MG |
|  | MG208975* (= KJ458539, MG208976) |  |
| *Monacha cantiana* CAN-1 Italian populations | **MG208977*** (= MG208978-MG208994), **MG208995*** | Pieńkowska et al. (2018b) MG |
| *Monacha cantiana* CAN-2 Italian populations | **MG208996*** (= MG208997), **MG208998*** (= MG208999-MG209004) | Pieńkowska et al. (2018b) |
| *Monacha cantiana* s.l. CAN-3 Italian populations | **MG209005*** (= MG209006), **MG209007*** | Pieńkowska et al. (2018b) |
| *Monacha cantiana* s.l. CAN-3 Austrian populations | **HQ204543*** (= KF596863) | Duda et al. (2011) & Kruckenhauser et al. (2014) HQ, Cadahia et al. (2014) KF |
|  | **MG209008*** (= MN100591, MN100595-MN100599), **MG209009*** (= MG209010, MN100590, MN100592-MN100594) | Pieńkowska et al. (2018b) MG, Pieńkowska et al. (2019a) MN |
| *Monacha cantiana* s.l. CAN-4  = *Monacha cemenelea* French populations | **MG209011*** (= MG209912-MG209914), **MG209015***, **MT952445*** | Pieńkowska et al. (2018b) MG, Čejka et al. (2020) MT |
| *Monacha cantiana* s.l. CAN-? Italian populations | **AY741419*** | Manganelli et al. (2005) AY |
| *Monacha cantiana* s.l. CAN-5 Italian populations | **MK066947*** (= MK066948-MK066950), **MK066951***, **MK066952*** (= MK066953, MK066956), **MK066954***, **MK066955***, **MK066957***, **MK066958***, **MK066959*** | Pieńkowska et al. (2019a) MK |
| *Monacha cantiana* s.l. CAN-6 Italian populations | **MK066960***, **MK066961***, **MK066962***, **MK066963*** (= MK066964) | Pieńkowska et al. (2019a) MK |
| *Monacha pantanellii* Italian populations | **MT376031*** (= MT376032-MT376033), **MT376034** (= MT376038-MT376039), **MT376035***, **MT376036***, **MT376037***, **MT376040*** (= MT376041-MT376044), **MT376045***, **MT376046*** (= MT376047-MT376048, MT376050, MT376056-MT376057, MT376076), **MT376051*** (= MT376052-MT376054, MT376058-MT376061), **MT376062*** (MT376063, MT376074), **MT376064*** (= MT376065-MT376066), **MT376067***, **MT376068***, MT376069* (= MT376070-MT376071, MT376083), **MT376072***, **MT376073***, **MT376075*** (= MT376080), **MT376077***, **MT376078***, **MT376079***, **MT378081*** (= MT376082), **MT376084***, **MT376085*** (= MT376086-MT376087) | Pieńkowska et al. (2020) |
| *Monacha parumcinta* Italian populations | **AY741418***, **MG209016***, **MG209017*** (= MG209018), **MG209019***, **MG209020***, **MG209021*** (MG209022-MG209024), **MG209025*** (= MG209026-MG209030) | Manganelli et al. (2005) AY, Pieńkowska et al. (2018b) MG |
| *Monacha cartusiana* Italian population | **KM247397***, **KX495378**** | Pieńkowska et al. (2015) KM, Neiber & Hausdorf (2017) KX, |
| *Monacha cartusiana* Spanish population | **KX495429**** | Neiber & Hausdorf (2017) |
| *Monacha cartusiana* Polish population | **KM247391*** | Pieńkowska et al. (2015) |
| *Monacha cartusiana* Hungarian population | KM247391* | Pieńkowska et al. (2015) KM |
| *Monacha cartusiana* Czech populations | KM247391* (= MT952393, MT952395), **MT952354***, **MT952392***, **MT952425****, **MT952426****, **MT952433****, **MT952434*** (= MT952435-MT952436), **MT952443****, **MT952444**** | Pieńkowska et al. (2015) KM, Čejka et al. (2020) MT |
| *Monacha cartusiana* French populations | **MT952354***, **ON350961**** | Čejka et al. (2020) MT, Pieńkowska et al. (2022) ON |
| *Trochulus hispidus* | **KX495398****, **KY818541****, **MG585431****, **MT755520*** | Neiber & Hausdorf (2017) KX, Neiber et al. (2017) KY, Caro et al. (2019) MG, Proćków et al. (2021) MT |

16SrDNA short * – 269-418 bp

16SrDNA long ** – 725-856 bp
