# Supplementary material for: ﻿Next step in Monachacantiana (Montagu, 1803) phylogeography: northern French and Dutch populations (Eupulmonata, Stylommatophora, Hygromiidae)
Source: Zookeys. 2024 Apr 23;1198:55–86. doi: 10.3897/zookeys.1198.119738 (PMC11061557; doi:10.3897/zookeys.1198.119738)
Supplement: Supplementary material 3 — H3 sequences from GenBank used for molecular analysis comparisons (haplotypes in bold) [file zookeys-1198-055_article-119738__-s003.docx]

**Table S3**. H3 sequences from GenBank used for molecular analysis comparisons (haplotypes in bold)

| **species** | **H3** | **references** |
| --- | --- | --- |
| *Monacha cantiana* CAN-1 UK populations | **MG209031** (= MG209032-MG209033), **MG209034**, **MG209035** (= MG209036), **MG209037**, **MG209038** | Pieńkowska et al. (2018b) MG |
| *Monacha cantiana* CAN-1 Italian populations | **MG209039** (= MG209040-MG209041), **MG209042** (= MG209043-MG209044), **MG209045**, **MG209046**, **MG209047**, **MG209048** (= MG209049), **MG209050** (= MG209051) | Pieńkowska et al. (2018b) MG |
| *Monacha cantiana* CAN-2 Italian populations | **MG209052** | Pieńkowska et al. (2018b) MG |
| *Monacha cantiana* s.l. CAN-3 Italian populations | **MG209053**, **MG209054** | Pieńkowska et al. (2018b) MG |
| *Monacha cantiana* s.l. CAN-4  = *Monacha cemenelea* French populations | **MG209058**, **MG209059** | Pieńkowska et al. (2018b) MG |
| *Monacha cantiana* s.l. CAN-5 Italian populations | **MK066965** (= MK066966-MK066967, MK066977), **MK066968** (= MK066970, MK066975-MK066976), **MK066969**, **MK066971, MK066972**, **MK066973**, **MK066974**, **MK066977** | Pieńkowska et al. (2019a) MK |
| *Monacha cantiana* s.l. CAN-6 Italian populations | **MK066978**, **MK066979**, **MK066980** | Pieńkowska et al. (2019a) MK |
| *Monacha pantanellii* Italian populations | **MT385776** (= MT385793, MT385804, MT385806, MT385810-MT385811, MT385814, MT385818-MT385820, MT385828), **MT385577** (= MT385778-MT385779), **MT385780**, **MT385781** (= MT385782), **MT385783**, **MT385784**, **MT385785** (= MT385788, MT385796), **MT385786** (= MT385787, MT385790, MT385800, MT385802, MT385807, MT385826), **MT385789**, **MT385791**, **MT385792** (= MT385795, MT385797, MT385799, MT385803, MT385812-MT385813, MT385816-MT385817, MT385821- MT385825, MT385827, MT385830, MT385832), **MT385794** (= MT385815), **MT385798** (= MT385805), **MT385801**, **MT385808**, **MT385809**, **MT385829, MT385831** | Pieńkowska et al. (2020) MT |
| *Monacha parumcinta* Italian populations | MG209061 (= MG209062-MG209067), **MG209068**, **MG209069**, **MG209070** | Pieńkowska et al. (2018b) MG |
| *Monacha cartusiana* Hungarian population | **MG209072** | Pieńkowska et al. (2018b) MG |
| *Monacha cartusiana* French populations | **ON325384** | Pieńkowska et al. (2022) ON |
| *Trochulus hispidus* | **MT758614** | Proćków et al. (2021) MT |
