# Supplementary material for: ﻿Next step in Monachacantiana (Montagu, 1803) phylogeography: northern French and Dutch populations (Eupulmonata, Stylommatophora, Hygromiidae)
Source: Zookeys. 2024 Apr 23;1198:55–86. doi: 10.3897/zookeys.1198.119738 (PMC11061557; doi:10.3897/zookeys.1198.119738)
Supplement: Supplementary material 4 — ITS2 sequences from GenBank used for molecular analysis comparisons (haplotypes in bold) [file zookeys-1198-055_article-119738__-s004.docx]

**Table S4**. ITS2 sequences from GenBank used for molecular analysis comparisons (haplotypes in bold)

| **species** | **(5.8SrDNA) + ITS2 + (28SrDNA)** | **references** |
| --- | --- | --- |
| *Monacha cantiana* CAN-1 Spanish population | KX495478 | Neiber & Hausdorf (2017) |
| *Monacha cartusiana* Italian population | **KX495431** | Neiber & Hausdorf (2017) KX |
| *Monacha cartusiana* Spanish populations | **KJ458625**, **KX495479**, **MG585447**, **MG585476** | Razkin et al. (2015) KJ, Neiber & Hausdorf (2017) KX, Caro et al. (2019) MG |
| *Monacha cartusiana* French population | **ON332790** | Pieńkowska et al. (2022) ON |
| *Trochulus hispidus* | **KY818647**, **KX495451**, **MG585474** | Neiber et al. (2017) KY, Neiber & Hausdorf (2017) KX, Caro et al. (2019) MG |
