# Supplementary material for: ﻿Next step in Monachacantiana (Montagu, 1803) phylogeography: northern French and Dutch populations (Eupulmonata, Stylommatophora, Hygromiidae)
Source: Zookeys. 2024 Apr 23;1198:55–86. doi: 10.3897/zookeys.1198.119738 (PMC11061557; doi:10.3897/zookeys.1198.119738)
Supplement: Supplementary material 5 — Concatenated sequences of COI+16SrDNA used in NJ/ML-MEGA7/IQ Tree/RAxML/BI analysis (Fig. 9) [file zookeys-1198-055_article-119738__-s005.docx]

**Table S5**. Concatenated sequences of COI+16SrDNA used in NJ/ML-MEGA7/IQ Tree/RAxML/BI analysis (Fig. 9).

COI sequences were 615 bp in length. Long 16SrDNA sequences were cut to 829 positions (the alignment of concatenated sequences COI and long 16SrDNA was then 1444 positions in length).

| **Concatenated sequence** | **COI haplotype** | **Long**  **16SrDNA haplotype** | **Locality and specimens (for number of locality and specimen acronyms see Table 1)** |
| --- | --- | --- | --- |
| *Monacha cantiana* CAN-1 | | | |
| COI16S 1 | COI 1 | 16S 1 | FR, Pas-de-Calais (1: Ard2; 2: Ble2; 4: Lic1, Lic5); NL, Veere (11: Vee1-2) |
| COI16S 2 | COI 1 | 16S 2 | FR, Pas-de-Calais (1: Ard4) |
| COI16S 3 | COI 1 | 16S 3 | FR, Pas-de-Calais (2: Ble1, Ble4; 3: Lar1; 4: Lic2); FR, Seine-Maritime (5: Bet1, Bet5; 6: Pie1, Pie4); FR, Somme (7: Epa2); FR, Oise (10: Fou1); NL, Veere (11: Vee1-3; 12: Vee2-4, Vee2-5); UK, Newcastle (16: New5) |
| COI16S 4 | COI 1 | 16S 4 | FR, Pas-de-Calais (3: Lar3, Lar5) |
| COI16S 5 | COI 1 | 16S 5 | FR, Pas-de-Calais (4: Lic4) |
| COI16S 6 | COI 1 | 16S 6 | FR, Seine-Maritime (5: Bet4) |
| COI16S 7 | COI 1 | 16S 7 | FR, Somme (7: Epa1) |
| COI16S 8 | COI 1 | 16S 9 | FR, Somme (7: Epa5) |
| COI16S 9 | COI 1 | 16S 10 | FR, Somme (8: Fro2) |
| COI16S 10 | COI 1 | 16S 11 | FR, Somme (8: Fro3) |
| COI16S 11 | COI 1 | 16S 12 | FR, Somme (8: Fro4) |
| COI16S 12 | COI 1 | 16S 13 | FR, Somme (8: Fro5) |
| COI16S 13 | COI 1 | 16S 14 | FR, Oise (9: Esc1, Esc3) |
| COI16S 14 | COI 1 | 16S 15 | FR, Oise (9: Esc5) |
| COI16S 15 | COI 1 | 16S 18 | FR, Oise (10: Fou5) |
| COI16S 16 | COI 1 | 16S 19 | NL, Veere (11: Vee1-1, Vee1-4; 12: Vee2-3) |
| COI16S 17 | COI 2 | 16S 4 | FR, Pas-de-Calais (3: Lar2) |
| COI16S 18 | COI 6 | 16S 16 | FR, Oise (10: Fou2) |
| COI16S 19 | COI 7 | 16S 3 | NL, Veere (11: Vee1-5) |
| COI16S 20 | COI 8 | 16S 3 | NL, Veere (12: Vee2-2) |
| COI16S 21 | COI 10 | 16S 20 | UK, Newcastle (16: New1, New4) |
| COI16S 22 | COI 11 | 16S 20 | UK, Newcastle (16: New3, New6) |
| COI16S 25 | MG208884 | 16S 1 | UK, Barrow (17: 8FG-1) |
| COI16S 26 | MG208885 | 16S 1 | UK, Barrow (17: 8FG-2) |
| COI16S 27 | MG208893 | 16S 1 | UK, Rotherham (18: Sit1-1) |
| COI16S 28 | MG208899 | 16S 21 | UK, Sheffield (19: Sit2-1) |
| COI16S 29 | MG208905 | 16S 24 | IT, Latium, Gole del Velino (20: 4FG-1) |
| COI16S 30 | MG208910 | 16S 25 | IT, Latium, Gole del Velino (20: 4FG-2) |
| COI16S 31 | MG208921 | 16S 26 | IT, Latium, Valle del Tronto (21: Tro-1) |
| COI16S 32 | MG208923 | 16S 27 | IT, Latium, Valle del Turano (22: Tur5-1) |
| COI16S 33 | MG208924 | 16S 28 | IT, Latium, Valle del Turano (22: Tur5-2) |
|  | KX507234 | KX495428 | SP, Pais Vasco, Sopelana(SP164 Neiber & Hausdorf, 2017) |
| *Monacha cantiana* CAN-2 | | | |
| COI16S 23 | COI 12 | 16S 22 | IT, Tuscany, Sasso di Simone (23: Sim1) |
| COI16S 24 | COI 12 | 16S 23 | IT, Tuscany, Sasso di Simone (23: Sim2) |
| COI16S 34 | MG208925 | 16S 29 | IT, Venetum, Sorgà (24: 12FG1) |
| COI16S 35 | MG208928 | 16S 30 | IT, Venetum, Sorgà (24: 12FG2) |
| *Monacha cantiana* s.l. CAN-3 | | | |
| COI16S 36 | MG208938 | 16S 31 | AU, Breitenlee (25: Dud2) |
| *Monacha cantiana* s.l. CAN-4 – *Monacha*) *cemenelea* | | | |
| COI16S 37 | MG208939 | 16S 32 | FR, Alpes-Maritimes (26: 3FG-1) |
| COI16S 38 | MG208940 | 16S 32 | FR, Alpes-Maritimes (26: 3FG-2) |
| *Monacha cartusiana* | | | |
|  | KX507189 | KX495378 | IT, Lombardia, Anfo towards Ponte Caffaro (ZMH 51710-1594 - Neiber & Hausdorf, 2017) |
|  | ON332653 | ON350961 | FR: Aude, Cubieres-sur-Cinoble (Cur-2 - Pieńkowska et al. 2022) |
| *Trochulus hispidus* | | | |
|  | KX507209 | KX495398 | DE, Hamburg (ZMH 119338-2410 – Neiber & Hausdorf, 2017) |
|  | KY818415 | KY818541 | AU, Upper Austria, Gmunden, Hallstatt (1820 – Neiber et al. 2017) |
|  | MT754799 | MT755459 | AU, Bodele (Bo3 - Proćków et al. 2021) |
