# Supplementary material for: ﻿Next step in Monachacantiana (Montagu, 1803) phylogeography: northern French and Dutch populations (Eupulmonata, Stylommatophora, Hygromiidae)
Source: Zookeys. 2024 Apr 23;1198:55–86. doi: 10.3897/zookeys.1198.119738 (PMC11061557; doi:10.3897/zookeys.1198.119738)
Supplement: Supplementary material 6 — Concatenated sequences of H3 + [(5.8SrDNA)+ITS2+(28SrDNA)] used in NJ/ML-MEGA7/IQ Tree/RAxML/BI analysis (Fig. 10) [file zookeys-1198-055_article-119738__-s006.docx]

**Table S6.** Concatenated sequences of H3 + [(5.8SrDNA)+ITS2+(28SrDNA)] in NJ/ML-MEGA7/IQ Tree/RAxML/BI analyses (Fig. 10).

H3 sequences were cut to 279 bp, 5.8SrDNA+ITS2+28SrDNA sequences were 775 positions in length (the concatenated sequences H3 + [(5.8SrDNA)+ITS2+(28SrDNA)] were then 1054 positions in length).

| **Concatenated sequence** | **H3 haplotype** | **ITS2 haplotype** | **Locality and specimens (for number of locality and specimen acronyms see Table 1)** |
| --- | --- | --- | --- |
| *Monacha cantiana* CAN-1 (French populations) | | | |
| H3ITS2 1 | H3 1 | ITS2 1 | FR, Pas-de-Calais (1: Ard1, Ard3, Ard4; 3: Lar3; 4: Lic5); FR, Seine-Maritime (6: Pie2); FR, Somme (7: Epa3; 8: Fro3); FR, Oise (10: Fou2); UK, Hurn (13: Hum1); UK, Vernhams Dean (14: Ver4); UK, Upton (15: Upt1, Upt2); UK, Newcastle (16: New4, New6) |
| H3ITS2 2 | H3 2 | ITS2 2 | FR, Pas-de-Calais (1: Ard2) |
| H3ITS2 3 | H3 1 | ITS2 3 | FR, Pas-de-Calais (2: Ble2) |
| H3ITS2 4 | H3 1 | ITS2 4 | FR, Pas-de-Calais (2: Ble5) |
| H3ITS2 5 | H3 3 | ITS2 5 | FR, Pas-de-Calais (3: Lar1) |
| H3ITS2 6 | H3 1 | ITS2 6 | FR, Pas-de-Calais (3: Lar2) |
| H3ITS2 7 | H3 4 | ITS2 7 | FR, Pas-de-Calais (3: Lar4) |
| H3ITS2 8 | H3 1 | ITS2 7 | FR, Pas-de-Calais (3: Lar5) |
| H3ITS2 9 | H3 1 | ITS2 8 | FR, Pas-de-Calais (4: Lic2) |
| H3ITS2 10 | H3 3 | ITS2 1 | FR, Pas-de-Calais (4: Lic3) |
| H3ITS2 11 | H3 1 | ITS2 9 | FR, Pas-de-Calais (4: Lic4) |
| H3ITS2 12 | H3 1 | ITS2 10 | FR, Seine-Maritime (5: Bet1) |
| H3ITS2 13 | H3 1 | ITS2 11 | FR, Seine-Maritime (5: Bet2) |
| H3ITS2 14 | H3 1 | ITS2 12 | FR, Seine-Maritime (5: Bet4) |
| H3ITS2 15 | H3 1 | ITS2 13 | FR, Seine-Maritime (5: Bet5) |
| H3ITS2 16 | H3 1 | ITS2 14 | FR, Seine-Maritime (6: Pie1) |
| H3ITS2 17 | H3 1 | ITS2 15 | FR, Seine-Maritime (6: Pie4) |
| H3ITS2 18 | H3 3 | ITS2 16 | FR, Somme (7: Epa1) |
| H3ITS2 19 | H3 1 | ITS2 17 | FR, Somme (7: Epa3) |
| H3ITS2 20 | H3 6 | ITS2 17 | FR, Oise (9: Esc5) |
| H3ITS2 21 | H3 5 | ITS2 18 | FR, Somme (7: Epa4) |
| H3ITS2 22 | H3 1 | ITS2 19 | FR, Somme (8: Fro2) |
| H3ITS2 23 | H3 1 | ITS2 20 | FR, Oise (9: Esc1) |
| H3ITS2 24 | H3 2 | ITS2 21 | FR, Oise (9: Esc2) |
| H3ITS2 25 | H3 6 | ITS2 22 | FR, Oise (9: Esc3) |
| H3ITS2 26 | H3 1 | ITS2 23 | FR, Oise (9: Esc4) |
| H3ITS2 27 | H3 3 | ITS2 24 | FR, Oise (10: Fou1) |
| H3ITS2 28 | H3 7 | ITS2 25 | FR, Oise (10: Fou4) |
| H3ITS2 29 | H3 1 | ITS2 26 | FR, Oise (10: Fou5) |
| *Monacha cantiana* CAN-1 (English populations) | | | |
| H3ITS2 30 | H3 9 | ITS2 1 | UK, Vernhams Dean (14: Ver1, Ver2); UK, Newcastle (16: New1, New2, New3, New5) |
| H3ITS2 31 | H3 10 | ITS2 27 | UK, Vernhams Dean (14: Ver3) |
| H3ITS2 32 | MG209031 | ITS2 1 | UK, Barrow (17: 8FG-1) |
| H3ITS2 33 | MG209032 | ITS2 1 | UK, Barrow (17: 8FG-2) |
| H3ITS2 34 | MG209038 | ITS2 1 | UK, Shefield (19: Sit2-1) |
| H3ITS2 35 | MG209035 | ITS2 28 | UK, Rotherham (18: Sit1-1) |
| *Monacha cantiana* CAN-1 (Italian populations) | | | |
| H3ITS2 36 | MG209039 | ITS2 29 | IT, Latium, Gole del Velino (20: 4FG1) |
| H3ITS2 37 | MG209042 | ITS2 29 | IT, Latium, Gole del Velino (20: 4FG2) |
| H3ITS2 38 | MG209043 | ITS2 1 | IT, Latium, Valle del Tronto (21: Tro1) |
| H3ITS2 39 | MG209048 | ITS2 29 | IT, Latium, Valle del Turano (22: Tur5-1) |
| *Monacha cantiana* CAN-2 | | | |
| H3ITS2 40 | MG209050 | ITS2 30 | IT, Venetum, Sorgà (24: 12FG1) |
| H3ITS2 41 | H3 1 | ITS2 31 | IT, Venetum, Sorgà (24: 12FG2) |
| *Monacha cantiana* s.l. CAN-3 | | | |
| H3ITS2 42 | MG209056 | ITS2 32 | AU, Breitenlee (25: Dud2) |
| *Monacha cantiana* s.l. CAN-4 – *Monacha* *cemenelea* | | | |
| H3ITS2 43 | MG209058 | ITS2 33 | FR, Alpes-Maritimes (26: 3FG-1) |
| H3ITS2 44 | MG209059 | ITS2 34 | FR, Alpes-Maritimes (26: 3FG-2) |
| *Trochulus hispidus* | | | |
|  | MT758614 | MG585474 | H3 AU, Bodele (Bo3 - Proćków et al. 2021), ITS2 SP (EHUMC-2078 – Caro et al. 2019) |
