# Supplementary material for: ﻿Next step in Monachacantiana (Montagu, 1803) phylogeography: northern French and Dutch populations (Eupulmonata, Stylommatophora, Hygromiidae)
Source: Zookeys. 2024 Apr 23;1198:55–86. doi: 10.3897/zookeys.1198.119738 (PMC11061557; doi:10.3897/zookeys.1198.119738)
Supplement: Supplementary material 7 — Concatenated sequences of COI + 16SrDNA long + H3 + [(5.8SrDNA)+ITS2+(28SrDNA)] used in NJ/ML-MEGA7/ML-IQ Tree/RAxML/BI analysis (Fig. 11) [file zookeys-1198-055_article-119738__-s007.docx]

**Table S7.** Concatenated sequences of COI + 16SrDNA long + H3 + [(5.8SrDNA)+ITS2+(28SrDNA)] in NJ/ML-MEGA7/IQ Tree/RAxML/BI analyses (Fig. 11). Length of the particular sequences were COI 615 bp, 16SrDNA - 829 bp, H3 - 279 bp, 5.8SrDNA+ITS2+28SrDNA - 775 bp (the concatenated sequences COI + 16SrDNA long + H3 + [(5.8SrDNA)+ITS2+(28SrDNA)] were then 2498 positions in length).

| **Concatenated sequence** | **COI haplotype** | **16SrDNA haplotype** | **H3 haplotype** | **ITS2 haplotype** | **Locality and specimens (for number of locality and specimen acronyms see Table 1)** |
| --- | --- | --- | --- | --- | --- |
| *Monacha cantiana* CAN-1 (French populations) | | | | | |
| CS 1 | COI 1 | 16S 1 | H3 2 | ITS2 2 | FR, Pas-de-Calais (1: Ard2) |
| CS 2 | COI 1 | 16S 2 | H3 1 | ITS2 1 | FR, Pas-de-Calais (1: Ard4) |
| CS 3 | COI 1 | 16S 1 | H3 1 | ITS2 3 | FR, Pas-de-Calais (2: Ble2) |
| CS 4 | COI 1 | 16S 3 | H3 3 | ITS2 5 | FR, Pas-de-Calais (3: Lar1) |
| CS 5 | COI 2 | 16S 4 | H3 1 | ITS2 6 | FR, Pas-de-Calais (3: Lar2) |
| CS 6 | COI 1 | 16S 4 | H3 1 | ITS2 1 | FR, Pas-de-Calais (3: Lar3) |
| CS 7 | COI 1 | 16S 4 | H3 1 | ITS2 7 | FR, Pas-de-Calais (3: Lar5) |
| CS 8 | COI 1 | 16S 3 | H3 1 | ITS2 8 | FR, Pas-de-Calais (4: Lic2) |
| CS 9 | COI 1 | 16S 5 | H3 1 | ITS2 9 | FR, Pas-de-Calais (4: Lic4) |
| CS 10 | COI 1 | 16S 1 | H3 1 | ITS2 1 | FR, Pas-de-Calais (4: Lic5) |
| CS 11 | COI 1 | 16S 3 | H3 1 | ITS2 10 | FR, Seine-Maritime (5: Bet1) |
| CS 12 | COI 1 | 16S 6 | H3 1 | ITS2 12 | FR, Seine-Maritime (5: Bet4) |
| CS 13 | COI 1 | 16S 3 | H3 1 | ITS2 13 | FR, Seine-Maritime (5: Bet5) |
| CS 14 | COI 1 | 16S 3 | H3 1 | ITS2 14 | FR, Seine-Maritime (6: Pie1) |
| CS 15 | COI 1 | 16S 3 | H3 1 | ITS2 15 | FR, Seine-Maritime (6: Pie4) |
| CS 16 | COI 1 | 16S 7 | H3 3 | ITS2 16 | FR, Somme (7: Epa1) |
| CS 17 | COI 1 | 16S 3 | H3 1 | ITS2 1 | FR, Somme (7: Epa2) |
| CS 18 | COI 1 | 16S 3 | H3 3 | ITS2 24 | FR, Oise (10: Fou1) |
| CS 19 | COI 1 | 16S 10 | H3 1 | ITS2 19 | FR, Somme (8: Fro2) |
| CS 20 | COI 1 | 16S 11 | H3 1 | ITS2 1 | FR, Somme (8: Fro3) |
| CS 21 | COI 1 | 16S 14 | H3 1 | ITS2 20 | FR, Oise (9: Esc1) |
| CS 22 | COI 1 | 16S 15 | H3 6 | ITS2 17 | FR, Oise (9: Esc5) |
| CS 23 | COI 1 | 16S 14 | H3 6 | ITS2 22 | FR, Oise (9: Esc3) |
| CS 24 | COI 6 | 16S 16 | H3 1 | ITS2 1 | FR, Oise (10: Fou2) |
| CS 25 | COI 1 | 16S 18 | H3 1 | ITS2 26 | FR, Oise (10: Fou5) |
| *Monacha cantiana* CAN-1 (English populations) | | | | | |
| CS 26 | COI 10 | 16S 20 | H3 9 | ITS2 1 | UK, Newcastle (16: New1) |
| CS 27 | COI 11 | 16S 20 | H3 9 | ITS2 1 | UK, Newcastle (16: New3) |
| CS 28 | COI 10 | 16S 20 | H3 1 | ITS2 1 | UK, Newcastle (16: New4) |
| CS 29 | COI 1 | 16S 3 | H3 9 | ITS2 1 | UK, Newcastle (16: New5) |
| CS 30 | COI 11 | 16S 20 | H3 1 | ITS2 1 | UK, Newcastle (16: New6) |
| CS 31 | MG208884 | 16S 1 | MG209031 | ITS2 1 | UK, Barrow (17: 8FG-1) |
| CS 32 | MG208885 | 16S 1 | MG209032 | ITS2 1 | UK, Barrow (17: 8FG-2) |
| CS 33 | MG208893 | 16S 1 | MG209035 | ITS2 28 | UK, Rotherham (18: Sit1-1) |
| CS 34 | MG208899 | 16S 21 | MG209038 | ITS2 1 | UK, Sheffield (19: Sit2-1) |
| *Monacha cantiana* CAN-1 (Italian populations) | | | | | |
| CS 35 | MG208905 | 16S 24 | MG209039 | ITS2 29 | IT, Latium, Gole del Velino (20: 4FG1) |
| CS 36 | MG208910 | 16S 25 | MG209042 | ITS2 29 | IT, Latium, Gole del Velino (20: 4FG2) |
| CS 37 | MG208921 | 16S 26 | MG209043 | ITS2 1 | IT, Latium, Valle del Tronto (21: Tro1) |
| CS 38 | MG208923 | 16S 27 | MG209048 | ITS2 29 | IT, Latium, Valle del Turano (22: Tur5-1) |
| *Monacha cantiana* CAN-2 (Italian population) | | | | | |
| CS 39 | MG208925 | 16S 29 | MG209050 | ITS2 30 | IT, Venetum, Sorgà (24: 12FG1) |
| CS 40 | MG208928 | 16S 30 | H3 1 | ITS2 31 | IT, Venetum, Sorgà (24: 12FG2) |
| *Monacha cantiana* s.l. CAN-3 (Austrian population) | | | | | |
| CS 41 | MG208938 | 16S 31 | MG209056 | ITS2 32 | AU, Breitenlee (25: Dud2) |
| *Monacha cantiana* s.l. CAN-4 (*Monacha cemenelea*) (French population) | | | | | |
| CS 42 | MG208939 | 16S 32 | MG209058 | ITS2 33 | FR, Alpes-Maritimes (26: 3FG-1) |
| CS 43 | MG208940 | 16S 32 | MG209059 | ITS2 34 | FR, Alpes-Maritimes (26: 3FG-2) |
| *Monacha cartusiana* (French population) | | | | | |
|  | ON332653 | ON350961 | ON325384 | ON332790 | FR, Occitania, Aude, Cubières-sur-Cinoble (Cur2) (Pieńkowska et al. 2020) |
| *Trochulus hispidus* | | | | | |
|  | KX507209 | KX495398 | MT758614 | KX495451 | DE, Hamburg (ZMH 119338-2410 – Neiber & Hausdorf, 2017) KX; AU, Bodele (Bo3 - Proćków et al. 2021) MT |
|  | KY818415 | KY818541 | MT758614 | KY818647 | AU, Upper Austria, Gmunden, Hallstatt (1820 – Neiber et al. 2017) KY; AU, Bodele (Bo3 - Proćków et al. 2021) MT |
|  | MG585398 | MG585431 | MT758614 | MG585474 | SP (EHUMC-2078 – Caro et al. 2020) MG; AU, Bodele (Bo3 - Proćków et al. 2021) MT |
